# Encapsulation of Crabtree's Catalyst in Sulfonated MIL‐101(Cr): Enhancement of Stability and Selectivity between Competing Reaction Pathways by the MOF Chemical Microenvironment

**DOI:** 10.1002/anie.201710091

**Published:** 2018-03-22

**Authors:** Alexios Grigoropoulos, Alasdair I. McKay, Alexandros P. Katsoulidis, Robert P. Davies, Anthony Haynes, Lee Brammer, Jianliang Xiao, Andrew S. Weller, Matthew J. Rosseinsky

**Affiliations:** ^1^ Department of Chemistry University of Liverpool Liverpool L69 7ZD UK; ^2^ Department of Chemistry University of Oxford Chemistry Research Laboratories Oxford OX1 3TA UK; ^3^ Department of Chemistry Imperial College London South Kensington London SW7 2AZ UK; ^4^ Department of Chemistry University of Sheffield Brook Hill Sheffield S3 7HF UK

**Keywords:** allylic alcohols, Crabtree's catalyst, encapsulation, hydrogenation, metal–organic frameworks

## Abstract

Crabtree's catalyst was encapsulated inside the pores of the sulfonated MIL‐101(Cr) metal–organic framework (MOF) by cation exchange. This hybrid catalyst is active for the heterogeneous hydrogenation of non‐functionalized alkenes either in solution or in the gas phase. Moreover, encapsulation inside a well‐defined hydrophilic microenvironment enhances catalyst stability and selectivity to hydrogenation over isomerization for substrates bearing ligating functionalities. Accordingly, the encapsulated catalyst significantly outperforms its homogeneous counterpart in the hydrogenation of olefinic alcohols in terms of overall conversion and selectivity, with the chemical microenvironment of the MOF host favouring one out of two competing reaction pathways.

Metal–organic frameworks (MOFs)^1^ are crystalline and permanently porous materials that have emerged as promising hosts for the immobilization of organometallic catalysts,^2^ since they allow control of the steric and chemical microenvironment around the encapsulated catalytically active species. This in turn could promote catalytic activity and selectivity through extended coordination sphere interactions. These concepts lie behind the exceptional reactivity and selectivity of metalloenzymes,^3^ however their transfer to the design and synthesis of artificial catalysts is challenging.^4^ Several examples of MOF‐supported catalysts showing exceptional overall catalytic activity have been reported.^5^, ^6^ Enhancement of selectivity between products of a single reaction pathway by control of the steric^7^ or the chemical^8^ microenvironment has also been demonstrated.

Crabtree's catalyst is one of the best commercially available homogeneous catalysts for hydrogenation of alkenes.^9^ However, it is deactivated in solution under hydrogenation conditions, forming catalytically inactive polymetallic hydride clusters.^10^ This self‐association reaction can be attenuated via modification of the coordination sphere of Ir^11^ or employment of larger weakly coordinating anions.^12^ Substrates bearing ligating functionalities such as olefinic alcohols show a more complicated behavior with Crabtree's catalyst since isomerization^13^ can also take place in parallel with hydrogenation.^14^

Here we use the Na^+^ salt of sulfonated MIL‐101(Cr) MOF (**1‐SO_3_Na**) to provide the anionic framework host for encapsulation of the cationic component of Crabtree's catalyst [Ir(cod)(PCy_3_)(py)][PF_6_] (**2‐PF_6_**) by cation exchange,^15^ forming **2@1‐SO_3_Na** (Scheme 1). Encapsulation of cation **2** inside a well‐defined, anionic and hydrophilic microenvironment forms an efficient heterogeneous catalyst for the hydrogenation of non‐functionalized alkenes in solution, enables hydrogenation in the gas phase, and most importantly enhances the catalyst's activity and selectivity for the hydrogenation of olefinic alcohols by suppressing the competing isomerization reaction. The MOF chemical microenvironment directs substrates along one of two distinct reaction pathways.

**Scheme 1:**
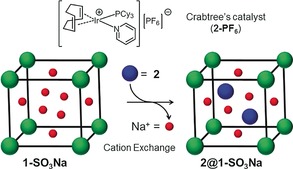
Encapsulation of the cationic component of Crabtree's catalyst (**2**, blue spheres) in sulfonated MIL‐101(Cr) (**1‐SO_3_Na**, cube) by exchange of the charge‐balancing Na^+^ cations (red spheres).

The sulfonated analogue of MIL‐101(Cr) (**1‐SO_3_H**)^16^ is a robust, readily synthesized anionic MOF. It is isostructural with pristine MIL‐101(Cr)^17^ with two charge‐balancing cations per formula unit, [H_*x*_Na_2−*x*_][Cr_3_(μ_3_‐O)(BDC‐SO_3_)_3_] (*x*=1.8±0.1, Figure S1, H_2_BDC‐SO_3_Na=2‐sulfoterephthalic acid sodium salt). Each cubic unit cell (*a=*87.63(3) Å) contains 8 bigger and 16 smaller mesopores, large enough to accommodate **2** (Figures S2 and S3). The cations within **1‐SO_3_H** can be partially exchanged with Ag^+[18]^ or [Rh(cod)(dppe)]^+^ [dppe=1,2‐bis(diphenylphosphino)ethane].^19^ To increase the number of exchangeable Na^+^ cations, **1‐SO_3_H** was treated with AcONa/AcOH buffer solution (pH 4.7), forming [H_*y*_Na_2−*y*_][Cr_3_(μ_3_‐O)(BDC‐SO_3_)_3_] (**1‐SO_3_Na**, *y=*0.2±0.1, Table S1).

Compound **1‐SO_3_Na** remains crystalline and mesoporous (Figures 1 a, b) with only a small change in the cubic unit cell parameter (*a*=87.99(4) Å) and a slight increase in the measured porosity (BET surface area=2005 m^2^ g^−1^, *V*_P_=0.91 cm^3^ g^−1^) and the pore size distribution, compared to **1‐SO_3_H** (Figure S9).

**Figure 1:**
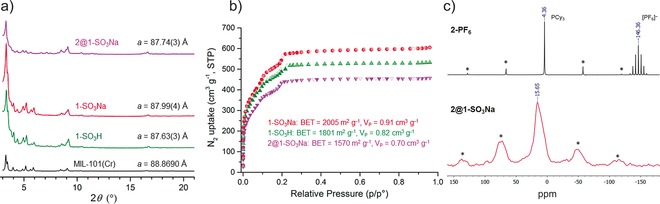
a) Comparison of PXRD patterns and unit cell parameter (*Fd* $\bar{3}$ *m* space group) for **2@1‐SO_3_Na** (magenta), **1‐SO_3_Na** (red), **1‐SO_3_H** (green) and **MIL‐101(Cr)** (calculated, black).^17^ Le Bail fits are included in the supporting information. b) N_2_ uptake of the desolvated materials at 77 K (BET=surface area, V_P_=pore volume). c) ^31^P{^1^H} MAS NMR spectrum of **2‐PF_6_** (black) and **2@1‐SO_3_Na** (red). Spinning side bands are marked with an asterisk.

After establishing an appropriate cation exchange protocol using [Cp*_2_Co]^+^ as a cationic probe (Table S2 and Figures S6, S9–S12), as we have shown previously,15b **2‐PF_6_** was used as a cationic guest precursor. Since water can poison the catalytically active species,^12^ cation exchange was carried out using desolvated **1‐SO_3_Na** as the anionic host in dry and degassed acetone, producing **2@1‐SO_3_Na**. Crystallinity and particle morphology were retained after cation exchange with only a minor change in the cubic unit cell parameter (*a*=87.74(3) Å, Figure 1 a, see Le Bail fit in Figure S7 and SEM images in Figures S11 and S12), whereas BET surface area (1570 m^2^ g^−1^) and pore volume (0.70 cm^3^ g^−1^) were reduced, compared to **1‐SO_3_Na** (Figure 1 b).

ICP‐OES after digestion of **2@1‐SO_3_Na** gave an Ir content of 2.28 wt %, indicating that 7 % of the Na^+^ cations have been exchanged with **2** (Table S3), which is close to the upper limit of about 9 % calculated by accounting for the guest‐accessible space of the host MOF and the size of the cationic guest (Figures S1–S3). ICP‐OES also showed an equimolar Ir/P ratio, and only one broad peak was observed (*δ*_P_=15.65, fwhm≈15 ppm) in the ^31^P{^1^H} MAS NMR spectrum of **2@1‐SO_3_Na**, assigned to the PCy_3_ ligand (Figure 1 c). Signals arising from the [PF_6_]^−^ anion were not observed either in the ^31^P{^1^H} MAS or the ^19^F{^1^H} solution NMR spectra of **2@1‐SO_3_Na** after digestion, in contrast with the respective spectra of **2‐PF_6_** (Figures 1 c and S13). The down‐field chemical shift and peak broadening observed for the signal due to the PCy_3_ ligand in the ^31^P{^1^H} MAS NMR spectrum of **2@1‐SO_3_Na**, compared to **2‐PF_6_**, likely originate from the different anionic environment surrounding **2**.^20^

The ^1^H solution NMR spectrum of **2@1‐SO_3_Na** after digestion showed three low intensity peaks at *δ*=8.22, 7.58 and 7.16 ppm, assigned to pyridine (Figure S14). Treatment of **2@1‐SO_3_Na** with D_2_ gas resulted in deuteration of the cod ligand and formation of [D_4_]‐cyclooctane, as detected by ^2^H MAS NMR spectroscopy (Figure S15). These analytical and spectroscopic data are consistent with cation **2** being encapsulated intact inside the mesopores of **1‐SO_3_Na** by a simple cation exchange process.

To explore the possible interaction of the sulfonate groups decorating the pore walls of **1‐SO_3_Na** with the Ir center of **2** after encapsulation, the tosylate anion [OTs]^−^ was selected to model the BDC‐SO_3_ linker. Two new complexes were synthesized, [Ir(cod)(PCy_3_)(py)][OTs] (**2‐OTs**) and [Ir(cod)(PCy_3_)(OTs)] (**3**), in which OTs^−^ acts as a counter anion or as a ligand to Ir, respectively (Figures 2 a,b, Figures S16, S17, Table S4). ^31^P{^1^H} and ^1^H EXSY NMR spectroscopy in CD_2_Cl_2_ (Figures S18–S20) revealed that a dynamic reversible ligand exchange takes place between complexes **2‐OTs** and **3**, with OTs replacing pyridine in the coordination sphere of Ir (Figure 2 c). This suggests that the sulfonate groups in **2@1‐SO_3_Na** may also play a non‐spectator role, with potential implications in catalysis, as discussed next.

**Figure 2:**
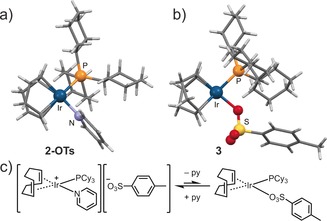
a) Single crystal structure of **2‐OTs** (OTs^−^ counter anion is not shown for clarity). b) Single crystal structure of **3**. c) Reversible ligand exchange between **2‐OTs** and **3** in CD_2_Cl_2_.

The catalytic performance of **2@1‐SO_3_Na** was benchmarked against **2‐PF_6_** in the hydrogenation of non‐functionalized alkenes in CH_2_Cl_2_ under mild conditions (Table 1). Control experiments verified that **1‐SO_3_Na** does not catalyze the hydrogenation of oct‐1‐ene (**4**). Introduction of **2@1‐SO_3_Na** as the catalyst afforded complete hydrogenation of **4** to *n*‐octane, at loadings as low as 50 ppm (entries 1–3). When the loading was reduced to 10 ppm (entry 4), conversion of **4** to *n*‐octane reached 83 % (TON=8.3×10^4^). Homogeneous catalyst **2‐PF_6_** under identical conditions produced comparable results, demonstrating that encapsulation is not detrimental to catalytic activity.

**Table 1:** Hydrogenation of non‐functionalized alkenes with heterogeneous **2@1‐SO_3_Na** and homogeneous **2‐PF_6_** catalysts.^[a]^ 
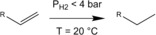

Entry	Substrate		Loading	*t*	**2@1‐SO_3_Na**		**2‐PF_6_**	
			[ppm]	[h]	Conv^[b]^	TON	Conv^[b]^	TON
					[%]		[%]	
1	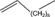	**4**	1000^[c]^	3	>99	>990	100	1000
2			100^[d]^	20	100	10 000	100	10 000
3			50^[e]^	24	100	20 000	–	–
4			10^[f]^	24	83	83 000	94	94 000
								
5	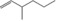	**5**	1000^[c]^	3	>99	>990	100	1000
6				20	100	1000	–	–
								
7	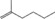	**6**	1000^[c]^	3	10	100	12	120
8				20	26	260	37	370
								
9	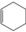	**7**	1000^[c]^	3	69	690	100	1000
10				20	81	810	–	–

[a] CH_2_Cl_2_ solvent, *T*=20 °C. [b] Conversion (%) based on GC. [c] [alkene]=0.5 m, *V*=1 mL, 8 mmol of H_2_. [d] [alkene]=1.0 m, *V*=4 mL, 16 mmol of H_2_. [e] [alkene]=1.0 m, *V*=10 mL, 48 mmol of H_2_. [f] [alkene]=1.5 m, *V*=12 mL, 48 mmol of H_2_.

The branched, but unhindered, aliphatic alkene, 3‐methylhex‐1‐ene (**5**) was also completely hydrogenated using **2@1‐SO_3_Na** at 1000 ppm loading (entries 5 and 6). The hindered aliphatic alkene, 2‐methylhex‐1‐ene (**6**) was only partially hydrogenated with either catalyst after 20 h (entries 7 and 8). Conversion did not increase any further after 72 h in either system, reflecting catalyst deactivation. When cyclohexene (**7**) was employed as a substrate, conversion reached 69 % in 3 h with **2@1‐SO_3_Na** as the catalyst but increased only to 81 % after 20 h. On the contrary, 100 % conversion was observed with **2‐PF_6_** in 3 h (entries 9 and 10).

The different response observed for this bulkier substrate is consistent with hydrogenation taking place within the pores and not on the surface of **2@1‐SO_3_Na**. The heterogeneity of the reaction was further established by carrying out a leaching test (Figure S21). Recycling of **2@1‐SO_3_Na** was also possible with a small decrease in activity (82 % conversion) during the third cycle (Figure S22).

Compound **2@1‐SO_3_Na** is a versatile catalyst which can also be employed in a gas/solid reaction,^21^ as demonstrated by the complete hydrogenation of but‐1‐ene over **2@1‐SO_3_Na** in 2.5 h (4000 μmol of but‐1‐ene hydrogenated per 1 mg of Ir). Although finely ground solid **2‐PF_6_** was also active, dispersion of **2** in the porous anionic solid‐state support increases the number of accessible catalytic sites in **2@1‐SO_3_Na**, resulting in a sixfold increase in activity compared to the non‐porous solid **2‐PF_6_** (Figure 3). Recycling of **2@1‐SO_3_Na** was also successful upon exposure to fresh but‐1‐ene (Figure S23).

**Figure 3:**
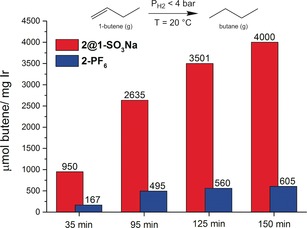
Conversion of but‐1‐ene into *n*‐butane in a gas/solid hydrogenation reaction over **2@1‐SO_3_Na** (red) and **2‐PF_6_** (blue). Conditions: *T*=20 °C, *P* ${}_{H{}_{2}}$ <4 bar, 0.5 mg of solid catalyst used.

The mesopores of **2@1‐SO_3_Na** are hydrophilic due to the presence of H‐bond accepting sulfonate groups as well as Lewis acidic Cr^III^ sites and Na^+^ cations. Therefore, the reactivity of Crabtree's catalyst with substrates bearing functional groups that can interact with such an environment could significantly change due to encapsulation. We chose to explore this by using olefinic alcohols as substrates, whose fundamental characteristic is the competition between hydrogenation and isomerization upon turnover.^22^ Hydrogenation of a series of olefinic alcohols was carried out under a ≈20‐fold excess of H_2_ (Table 2).

**Table 2:** Substrate conversion^[a]^ and product selectivity^[a,b]^ for hydrogenation of olefinic alcohols with heterogeneous **2@1‐SO_3_Na** and homogeneous **2‐PF_6_** catalysts.^[c]^ 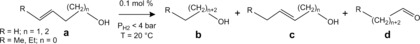

Entry	Substrate		*t*	**2@1‐SO_3_Na**				**2‐PF_6_**			
			[h]	Conv [%]	**b**	**c**	**d**	Conv [%]	**b**	**c**	**d**
1	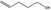	**8 a**	3	34	100	n.d.^[d]^	n.d.	100	100	n.d.	n.d.
2			24	100	100	n.d.	n.d.	–	–	–	–
											
3	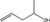	**9 a**	3	22	100	n.d.	n.d.	100	100	n.d.	n.d.
4			24	100	100	n.d.	n.d.	–	–	–	–
											
5	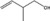	**10 a**	3	10	100	n.d.	n.d.	100	100	n.d.	n.d.
6			24	100	100	n.d.	n.d.	–	–	–	–
											
7	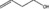	**11 a**	3	33	95	5	n.d.	56	85	13	2
8			24	100	100	n.d.	n.d.	57	86	12	2
											
9	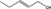	**12 a**	3	41	93	n.d.	7	69^[e]^	61	n.d.	35
10			24	96	92	n.d.	8	62^[e]^	55	n.d.	19
											
11	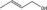	**13 a**	3	26	92	n.d.	8	54^[e]^	31	n.d.	54
12			24	82	90	n.d.	10	53^[e]^	28	n.d.	26

[a] Based on ^1^H NMR using mesitylene as standard for verifying mass‐balance. [b] Yield of each product over total conversion. [c] 0.1 mol % loading, [substrate]=0.5 m in CH_2_Cl_2_, *V*=0.7 mL, ≈8 mmol of H_2_. [d] Not detected. [e] Formation of ill‐defined condensation products was also observed, especially in 24 h.

Complete hydrogenation of pent‐4‐en‐1‐ol (**8 a**), pent‐4‐en‐2‐ol (**9 a**), and 2‐methylbut‐3‐en‐1‐ol (**10 a**) to the respective alcohols **8 b**–**10 b** was observed with **2‐PF_6_** in 3 h. Isomerization products were not detected (Figure S24), as reported for **2‐PF_6_** using similar substrates.^23^ Complete hydrogenation of **8 a**–**10 a** to **8 b**–**10 b** was also achieved with **2@1‐SO_3_Na**, albeit in 24 h (Table 2, entries 1–6). Isomerization products were again not detected. Conversion in 3 h correlates well with the steric hindrance around the double bond of the substrate: 10 % for **10 a** (more hindered), increasing to 22 % for **9 a** (less hindered), and reaching 34 % for **8 a** (linear). Olefinic alcohols **8 a**–**10 a** were hydrogenated considerably slower with **2@1‐SO_3_Na**, compared to the sterically comparable non‐functionalized alkenes **4** and **5** (Table 1). This is consistent with a strong interaction between the hydroxyl group of the olefinic alcohols and the chemical microenvironment of **2@1‐SO_3_Na**.

Substrates which are intrinsically more susceptible to isomerization, such as the homoallylic (**11 a**) and allylic (**12 a**, **13 a**) alcohols,^23^, ^24^ revealed a significant enhancement of reactivity and selectivity to hydrogenation with **2@1‐SO_3_Na**, compared to its homogeneous counterpart. The homogeneous catalyst **2‐PF_6_** afforded 56 % conversion of **11 a** in 3 h and 57 % in 24 h, indicative of catalyst deactivation (Table 2, entries 7 and 8, Figure S25). Moreover, isomerization of **11 a** was also observed, producing a non‐negligible amount of the internal olefinic alcohol **11 c** and traces of the aldehyde **11 d**. As a result, selectivity to hydrogenation and formation of *n*‐butanol (**11 b**) was only 86 % for the homogeneous system.

By contrast, the heterogeneous catalyst **2@1‐SO_3_Na** afforded complete conversion and 100 % selectivity to hydrogenation and formation of **11 b** (Table 2, entries 7 and 8, Figure S26). Monitoring conversion over time for both systems (Figure S27) verified that **2‐PF_6_** is deactivated after 3 h, whereas **2@1‐SO_3_Na** remained productive, affording full conversion in 6 h. Although traces of the internal olefin **11 c** were detected in short reaction times, **11 c** was subsequently also hydrogenated to **11 b**. The encapsulated catalyst is thus more stable, more active with respect to overall conversion, and more selective.

The superior performance of **2@1‐SO_3_Na** was even more pronounced in the hydrogenation of allylic alcohols that can isomerize directly to the respective aldehydes. Conversion under hydrogenation conditions for *trans*‐pent‐2‐en‐1‐ol (**12 a**, entries 9 and 10) and *trans*‐crotyl alcohol (**13 a**, entries 11 and 12) in 3 h with **2‐PF_6_** was 69 % and 54 %, respectively (Figure S28). Conversion did not increase after 24 h, indicating catalyst deactivation. Selectivity to hydrogenation was poor: 61 % for alcohol **12 b** in 3 h with a substantial amount of the aldehyde **12 d** formed (35 % selectivity), and 31 % for alcohol **13 b** in 3 h with the aldehyde **13 d** now being the main product (54 % selectivity). By contrast, overall conversion with **2@1‐SO_3_Na** as the catalyst reached 96 % for **12 a** and 82 % for **13 a** in 24 h (Figure S29). Isomerization to the aldehydes **12 d** and **13 d** was significantly suppressed, resulting in ≥90 % selectivity for the alcohols **12 b** and **13 b**.

To probe the effect of the sulfonate group on stability and selectivity, we also investigated the homogeneous hydrogenation of crotyl alcohol using **2‐OTs** and **3** as catalysts (Figure S30). Higher conversions were observed compared to **2‐PF_6_** (77 % for **2‐OTs** and 83 % for **3** in 24 h) in accordance with OTs^−^ being a more strongly coordinating anion, hence prolonging the catalyst's lifetime.^25^ By contrast, selectivity to hydrogenation did not significantly improve (39 % for **2‐OTs** and 53 % for **3**), remaining considerably lower than that of **2@1‐SO_3_Na** (≥90 %).

The reaction pathways for the hydrogenation or isomerization of olefinic alcohols with the homogeneous catalyst **2‐PF_6_** likely share the same starting point, the formation of a cationic Ir^III^‐dihydride complex in which the hydroxyl group is also coordinated to Ir (Scheme 2, intermediate **I**), followed by migratory insertion (intermediate **II**).^13^, ^14^ Bifurcation into separate, competitive pathways then occurs: i) hydrogenation to the respective alcohol via reductive elimination (pathway **A**) or ii) isomerization to the internal olefin via β‐elimination, which requires an appropriately orientated vacant coordination site, followed by off‐cycle tautomerization to the aldehyde (pathway **B**).

**Scheme 2:**
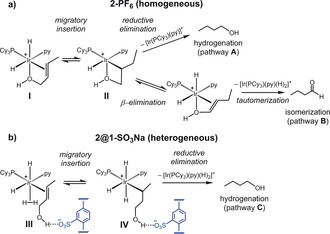
a) Competing pathways for hydrogenation (**A**) and isomerization (**B**) of olefinic alcohols with **2‐PF_6_**. b) Proposed pathway for hydrogenation (**C**) and suppression of isomerization with **2@1‐SO_3_Na**.

The significantly improved selectivity to hydrogenation observed with **2@1‐SO_3_Na** suggests that isomerization is suppressed. We propose that this could take place due to extended coordination sphere interactions between the hydroxyl group of the olefinic alcohols and the chemical microenvironment around **2**, such as H‐bonding to the sulfonate groups. This disfavors coordination of the hydroxyl group to Ir and enables formation of the dihydrogen complex **III**, in preference to **I** (pathway **C**). Productive hydrogenation occurs via an octahedral Ir^V^‐trihydride species (**IV**), as proposed for non‐functionalized alkenes with Crabtree‐type catalysts^26^ and β‐elimination is suppressed since Ir is coordinatively saturated throughout.

Catalyst **2@1‐SO_3_Na** also resulted in higher overall conversions for the hydrogenation of olefinic alcohols, compared to **2‐PF_6_**. A series of selective poisoning experiments revealed that the isomerization products are not responsible for catalyst deactivation (Table S7). We thus suggest that **2@1‐SO_3_Na** has a longer lifetime due to: i) spatial isolation of the positively charged catalytically active species inside the pores of the anionic MOF which hinders the formation of catalytically inactive clusters and/or ii) reversible coordination of the sulfonate anion, as shown with **2‐OTs** and **3**.

In summary, we demonstrate that the hybrid catalyst **2@1‐SO_3_Na** is capable of hydrogenating non‐functionalized alkenes at low loadings in solution and in the gas phase under mild conditions. It outperforms its homogeneous counterpart in the hydrogenation of olefinic alcohols, showing significantly higher conversions under otherwise identical conditions. In addition, encapsulation results in a pronounced selectivity enhancement in favor of hydrogenation by suppressing the competing isomerization reaction due to extended coordination sphere interactions of the catalytic center with the chemically functionalized internal surface of the MOF. Capitalizing on such stability and selectivity enhancements is likely to be important in catalytic applications in continuous flow.^27^ In metalloenzymes, it is well‐established that well‐positioned amino acid residues around the active site control reactivity and selectivity.^3^ Here, the well‐defined, readily engineered MOF chemical microenvironment controls reactivity and selectivity of the encapsulated catalyst, allowing discrimination between two distinct reaction pathways.

## Conflict of interest

The authors declare no conflict of interest.

## Supporting information

As a service to our authors and readers, this journal provides supporting information supplied by the authors. Such materials are peer reviewed and may be re‐organized for online delivery, but are not copy‐edited or typeset. Technical support issues arising from supporting information (other than missing files) should be addressed to the authors.

Supplementary

## References

[1a] H. Furukawa , K. E. Cordova , M. O'Keeffe , O. M. Yaghi , Science 2013, 341, 1230444; 23990564 10.1126/science.1230444

[1b] B. Li , H.-M. Wen , Y. Cui , W. Zhou , G. Qian , B. Chen , Adv. Mater. 2016, 28, 8819–8860; 27454668 10.1002/adma.201601133

[1c] H.-C. Zhou , S. Kitagawa , Chem. Soc. Rev. 2014, 43, 5415–5418, and references therein. 25011480 10.1039/c4cs90059f

[2a] J. Gascon , A. Corma , F. Kapteijn , F. X. Llabrés i Xamena , ACS Catal. 2014, 4, 361–378;

[2b] A. H. Chughtai , N. Ahmad , H. A. Younus , A. Laypkov , F. Verpoort , Chem. Soc. Rev. 2015, 44, 6804–6849; 25958955 10.1039/c4cs00395k

[2c] A. Grigoropoulos in Modern Developments in Catalysis, World Scientific (Europe), London, 2016, pp. 123–158;

[2d] S. M. J. Rogge , A. Bavykina , J. Hajek , H. Garcia , A. I. Olivos-Suarez , A. Sepulveda-Escribano , A. Vimont , G. Clet , P. Bazin , F. Kapteijn , M. Daturi , E. V. Ramos-Fernandez , F. X. Llabres i Xamena , V. Van Speybroeck , J. Gascon , Chem. Soc. Rev. 2017, 46, 3134–3184. 28338128 10.1039/c7cs00033b PMC5708534

[3a] S. W. Ragsdale , Chem. Rev. 2006, 106, 3317–3337; 16895330 10.1021/cr0503153

[3b] M. Zhao , H.-B. Wang , L.-N. Ji , Z.-W. Mao , Chem. Soc. Rev. 2013, 42, 8360–8375; 23881282 10.1039/c3cs60162e

[3c] R. H. Holm , E. I. Solomon , Chem. Rev. 2014, 114, 3367–3368, and references therein. 24712923 10.1021/cr500118g

[4a] C. W. Jones , Top. Catal. 2010, 53, 942–952;

[4b] M. Raynal , P. Ballester , A. Vidal-Ferran , P. W. N. M. van Leeuwen , Chem. Soc. Rev. 2014, 43, 1660–1733 and 1734–1787; 24356298 10.1039/c3cs60027k

[4c] C. J. Brown , F. D. Toste , R. G. Bergman , K. N. Raymond , Chem. Rev. 2015, 115, 3012–3035; 25898212 10.1021/cr4001226

[4d] S. Hübner , J. G. de Vries , V. Farina , Adv. Synth. Catal. 2016, 358, 3–25.

[5a] A. Fateeva , P. A. Chater , C. P. Ireland , A. A. Tahir , Y. Z. Khimyak , P. V. Wiper , J. R. Darwent , M. J. Rosseinsky , Angew. Chem. Int. Ed. 2012, 51, 7440–7444; 10.1002/anie.201202471 22696508

[5b] L. Mitchell , P. Williamson , B. Ehrlichová , A. E. Anderson , V. R. Seymour , S. E. Ashbrook , N. Acerbi , L. M. Daniels , R. I. Walton , M. L. Clarke , P. A. Wright , Chem. Eur. J. 2014, 20, 17185–17197; 25348903 10.1002/chem.201404377

[5c] K. Manna , T. Zhang , M. Carboni , C. W. Abney , W. Lin , J. Am. Chem. Soc. 2014, 136, 13182–13185; 25187995 10.1021/ja507947d

[5d] H. Fei , S. M. Cohen , J. Am. Chem. Soc. 2015, 137, 2191–2194; 25650584 10.1021/ja5126885

[5e] Z. Li , J.-D. Xiao , H.-L. Jiang , ACS Catal. 2016, 6, 5359–5365;

[5f] A. M. Fracaroli , P. Siman , D. A. Nagib , M. Suzuki , H. Furukawa , F. D. Toste , O. M. Yaghi , J. Am. Chem. Soc. 2016, 138, 8352–8355; 27346625 10.1021/jacs.6b04204 PMC5376101

[5g] N. C. Thacker , Z. Lin , T. Zhang , J. C. Gilhula , C. W. Abney , W. Lin , J. Am. Chem. Soc. 2016, 138, 3501–3509. 26885768 10.1021/jacs.5b13394

[6a] J. Canivet , S. Aguado , Y. Schuurman , D. Farrusseng , J. Am. Chem. Soc. 2013, 135, 4195–4198; 23470137 10.1021/ja312120x

[6b] A. M. Rasero-Almansa , A. Corma , M. Iglesias , F. Sanchez , Green Chem. 2014, 16, 3522–3527;

[6c] S. A. Burgess , A. Kassie , S. A. Baranowski , K. J. Fritzsching , K. Schmidt-Rohr , C. M. Brown , C. R. Wade , J. Am. Chem. Soc. 2016, 138, 1780–1783; 26813149 10.1021/jacs.5b12366

[6d] M. Rimoldi , A. Nakamura , N. A. Vermeulen , J. J. Henkelis , A. K. Blackburn , J. T. Hupp , J. F. Stoddart , O. K. Farha , Chem. Sci. 2016, 7, 4980–4984; 10.1039/c6sc01376g PMC6018438 30155148

[6e] A. Burgun , C. J. Coghlan , D. M. Huang , W. Chen , S. Horike , S. Kitagawa , J. F. Alvino , G. F. Metha , C. J. Sumby , C. J. Doonan , Angew. Chem. Int. Ed. 2017, 56, 8412–8416; 10.1002/anie.201611254 28160366

[7a] Z. Guo , C. Xiao , R. V. Maligal-Ganesh , L. Zhou , T. W. Goh , X. Li , D. Tesfagaber , A. Thiel , W. Huang , ACS Catal. 2014, 4, 1340–1348;

[7b] S. Yuan , Y. P. Chen , J. S. Qin , W. Lu , L. Zou , Q. Zhang , X. Wang , X. Sun , H.-C. Zhou , J. Am. Chem. Soc. 2016, 138, 8912–8919; 27345035 10.1021/jacs.6b04501

[7c] L. Liu , T.-Y. Zhou , S. G. Telfer , J. Am. Chem. Soc. 2017, 139, 13936–13943. 28929762 10.1021/jacs.7b07921

[8a] D. J. Xiao , J. Oktawiec , P. J. Milner , J. R. Long , J. Am. Chem. Soc. 2016, 138, 14371–14379; 27704846 10.1021/jacs.6b08417

[8b] W. Shi , L. Cao , H. Zhang , X. Zhou , B. An , Z. Lin , R. Dai , J. Li , C. Wang , W. Lin , Angew. Chem. Int. Ed. 2017, 56, 9704–9709; 10.1002/anie.201703675 28543992

[8c] L. Li , Q. Yang , S. Chen , X. Hou , B. Liu , J. Lu , H.-L. Jiang , Chem. Commun. 2017, 53, 10026–10029. 10.1039/c7cc06166h 28836636

[9] R. Crabtree , Acc. Chem. Res. 1979, 12, 331–337.

[10a] D. F. Chodosh , R. H. Crabtree , H. Felkin , S. Morehouse , G. E. Morris , Inorg. Chem. 1982, 21, 1307–1311;

[10b] Y. Xu , M. A. Celik , A. L. Thompson , H. Cai , M. Yurtsever , B. Odell , J. C. Green , D. M. P. Mingos , J. M. Brown , Angew. Chem. Int. Ed. 2009, 48, 582–585; 10.1002/anie.200804484 19067451

[11a] H. M. Lee , T. Jiang , E. D. Stevens , S. P. Nolan , Organometallics 2001, 20, 1255–1258;

[11b] L. D. Vazquez-Serrano , B. T. Owens , J. M. Buriak , Inorg. Chim. Acta 2006, 359, 2786–2797;

[11c] E. L. Kolychev , S. Kronig , K. Brandhorst , M. Freytag , P. G. Jones , M. Tamm , J. Am. Chem. Soc. 2013, 135, 12448–12459. 23883399 10.1021/ja406529c

[12a] S. P. Smidt , N. Zimmermann , M. Studer , A. Pfaltz , Chem. Eur. J. 2004, 10, 4685–4693; 15372652 10.1002/chem.200400284

[12b] G. L. Moxham , T. M. Douglas , S. K. Brayshaw , G. Kociok-Kohn , J. P. Lowe , A. S. Weller , Dalton Trans. 2006, 5492–5505. 17117219 10.1039/b612049k

[13] H. Li , C. Mazet , Acc. Chem. Res. 2016, 49, 1232–1241. 27159335 10.1021/acs.accounts.6b00144

[14a] G. Stork , D. E. Kahne , J. Am. Chem. Soc. 1983, 105, 1072–1073;

[14b] R. H. Crabtree , M. W. Davis , J. Org. Chem. 1986, 51, 2655–2661.

[15a] D. T. Genna , A. G. Wong-Foy , A. J. Matzger , M. S. Sanford , J. Am. Chem. Soc. 2013, 135, 10586–10589; 23837970 10.1021/ja402577s

[15b] A. Grigoropoulos , G. F. S. Whitehead , N. Perret , A. P. Katsoulidis , F. M. Chadwick , R. P. Davies , A. Haynes , L. Brammer , A. S. Weller , J. Xiao , M. J. Rosseinsky , Chem. Sci. 2016, 7, 2037–2050. 10.1039/c5sc03494a PMC5968521 29899929

[16a] G. Akiyama , R. Matsuda , H. Sato , M. Takata , S. Kitagawa , Adv. Mater. 2011, 23, 3294–3297; 21661069 10.1002/adma.201101356

[16b] Y.-X. Zhou , Y.-Z. Chen , Y. Hu , G. Huang , S.-H. Yu , H.-L. Jiang , Chem. Eur. J. 2014, 20, 14976–14980; 25291973 10.1002/chem.201404104

[16c] J. Juan-Alcañiz , R. Gielisse , A. B. Lago , E. V. Ramos-Fernandez , P. Serra-Crespo , T. Devic , N. Guillou , C. Serre , F. Kapteijn , J. Gascon , Catal. Sci. Technol. 2013, 3, 2311–2318.

[17] G. Férey , C. Mellot-Draznieks , C. Serre , F. Millange , J. Dutour , S. Surblé , I. Margiolaki , Science 2005, 309, 2040–2042. 16179475 10.1126/science.1116275

[18] Y. Zhang , B. Li , R. Krishna , Z. Wu , D. Ma , Z. Shi , T. Pham , K. Forrest , B. Space , S. Ma , Chem. Commun. 2015, 51, 2714–2717. 10.1039/c4cc09774b 25575193

[19] D. T. Genna , L. Y. Pfund , D. C. Samblanet , A. G. Wong-Foy , A. J. Matzger , M. S. Sanford , ACS Catal. 2016, 6, 3569–3574.

[20] Note: Although we cannot discount chemical shift changes in the ^31^P SS NMR spectrum due to the presence of paramagnetic Cr^III^ centers, no significant changes were reported when molecular species were incorporated into pristine MIL-101(Cr). See reference [17].

[21a] D. Yang , S. O. Odoh , J. Borycz , T. C. Wang , O. K. Farha , J. T. Hupp , C. J. Cramer , L. Gagliardi , B. C. Gates , ACS Catal. 2016, 6, 235–247;

[21b] F. M. Chadwick , A. I. McKay , A. J. Martinez-Martinez , N. H. Rees , T. Kramer , S. A. Macgregor , A. S. Weller , Chem. Sci. 2017, 8, 6014–6029. 28989631 10.1039/c7sc01491k PMC5625289

[22a] M. Moreno , L. N. Kissell , J. B. Jasinski , F. P. Zamborini , ACS Catal. 2012, 2, 2602–2613;

[22b] E. Sadeghmoghaddam , H. Gu , Y.-S. Shon , ACS Catal. 2012, 2, 1838–1845. 27642537 10.1021/cs300270d PMC5025262

[23a] L. Mantilli , C. Mazet , Tetrahedron Lett. 2009, 50, 4141–4144;

[23b] L. Mantilli , D. Gérard , S. Torche , C. Besnard , C. Mazet , Angew. Chem. Int. Ed. 2009, 48, 5143–5147; 10.1002/anie.200901863 19526480

[24] R. Uma , C. Crevisy , R. Gree , Chem. Rev. 2003, 103, 27–51. 12517180 10.1021/cr0103165

[25a] A. Rifat , G. Kociok-Köhn , J. W. Steed , A. S. Weller , Organometallics 2004, 23, 428–432;

[25b] E. Piras , F. Läng , H. Rüegger , D. Stein , M. Wörle , H. Grützmacher , Chem. Eur. J. 2006, 12, 5849–5858. 16718725 10.1002/chem.200501470

[26] J. J. Verendel , O. Pàmies , M. Diéguez , P. G. Andersson , Chem. Rev. 2014, 114, 2130–2169. 24568181 10.1021/cr400037u

[27] U. Hintermair , G. Francio , W. Leitner , Chem. Commun. 2011, 47, 3691–3701. 10.1039/c0cc04958a 21270995

