# Supplementary material for: Encapsulation of Crabtree's Catalyst in Sulfonated MIL‐101(Cr): Enhancement of Stability and Selectivity between Competing Reaction Pathways by the MOF Chemical Microenvironment
Source: Angew Chem Int Ed Engl. 2018 Mar 22;57(17):4532–7. doi: 10.1002/anie.201710091 (PMC5947555; doi:10.1002/anie.201710091)
Supplement: Supplementary file 1 — Supplementary [file ANIE-57-4532-s001.pdf]

Angewandte Chemie
Eine Zeitschrift der Gesellschaft Deutscher Chemiker

Supporting Information

Encapsulation of Crabtree's Catalyst in Sulfonated MIL-101(Cr): Enhancement of Stability and Selectivity between Competing Reaction Pathways by the MOF Chemical Microenvironment

Alexios Grigoropoulos, Alasdair I. McKay, Alexandros P. Katsoulidis, Robert P. Davies, Anthony Haynes, Lee Brammer, Jianliang Xiao, Andrew S. Weller, and Matthew J. Rosseinsky**

anie_201710091_sm_miscellaneous_information.pdf

Author Contributions

A.G. Writing—original draft: Lead; Writing—review & editing: Equal; Materials synthesis and characterization, catalysis: Lead
A.M. Writing—review & editing: Equal; Synthesis of complexes, crystallography and NMR spectroscopy: Lead
A.K. Writing—review & editing: Supporting
R.D. Writing—review & editing: Equal
A.H. Writing—review & editing: Equal
L.B. Writing—review & editing: Equal
J.X. Writing—review & editing: Equal
A.W. Writing—review & editing: Equal
M.R. Writing—review & editing: Equal.

Table of Contents

**1.** Materials, methods and instrumentation	S4
**2.** NMR spectroscopy	S5
**3.** X-ray diffraction	S6
**4.** Synthesis	S7
**5.** Catalysis	S11
**Table S1.** ICP-OES and CHN-S elemental analysis for **1-SO_3H** and **1-SO_3Na**	S15
**Table S2.** ICP-OES and CHN-S elemental analysis for **$[Cp*_2Co]^+$@1-SO_3Na**	S16
**Table S3.** ICP-OES analysis for **2@1-SO_3Na**	S16
**Table S4.** Selected bond lengths (Å) and angles (°) for **2-PF_6**, **2-OTs** and **3**	S17
**Table S5.** Crystallographic details	S18
**Table S6.** GC retention time of alkenes and alcohols	S19
**Table S7.** Selective poisoning experiments	S20
**Figure S1.** Synthesis and structural model of **1-SO_3H**	S21
**Figure S2.** Pore windows of MIL-101(Cr) and isostructural **1-SO_3H**	S21
**Figure S3.** Single crystal structure and dimensions of **2-PF_6**	S22
**Figure S4.** Le Bail fit of the PXRD pattern of **1-SO_3H**	S23
**Figure S5.** Le Bail fit of the PXRD pattern of **1-SO_3Na**	S23
**Figure S6.** Le Bail fit of the PXRD pattern of **$[Cp*_2Co]^+$@1-SO_3Na**	S24
**Figure S7.** Le Bail fit of the PXRD pattern of **2@1-SO_3Na**	S24
**Figure S8.** TGA graphs for **1-SO_3H** and **1-SO_3Na**	S25
**Figure S9.** PXRD patterns, N_2 uptake isotherms and pore size distribution	S26
**Figure S10.** ^1H NMR spectrum of **$[Cp*_2Co]^+$@1-SO_3Na** in 0.5 M NaOD	S27
**Figure S11.** SEM images for **1-SO_3H** and **1-SO_3Na**	S28
**Figure S12.** SEM images for **$[Cp*_2Co]^+$@1-SO_3Na** and **2@1-SO_3Na**	S29
**Figure S13.** ^1H, ^{19}F and ^{31}P NMR spectra of **2-PF_6** in CD_2Cl_2	S28
**Figure S14.** ^1H NMR spectrum of **2@1-SO_3Na** in 0.5 M NaOD	S29
**Figure S15.** ^2H MAS NMR spectrum of **2-PF_6** and **2@1-SO_3Na** after deuteration	S32
**Figure S16.** Single crystal structure of **2-OTs**	S33
**Figure S17.** Single crystal structure of **3**	S34
**Figure S18.** ^1H NMR spectra of **2-OTs** and **3** in CD_2Cl_2	S35
**Figure S19.** ^{31}P NMR spectra of **2-OTs** and **3** in CD_2Cl_2	S36
**Figure S20.** ^1H EXSY NMR spectrum of **2-OTs** in CD_2Cl_2	S37
**Figure S21.** Leaching test with **2@1-SO_3Na** as the catalyst	S38

Figure S22. Recycling of **2@1-SO$_3$Na** with 1-octene S38
Figure S23. Gas/solid hydrogenation of 1-butene S39
Figure S24. ^{1}H NMR spectra for hydrogenation of pent-4-en-1-ol (**8a**) S40
Figure S25. ^{1}H NMR spectra for homogeneous hydrogenation of but-3-en-1-ol (**11a**) S41
Figure S26. ^{1}H NMR spectra for heterogeneous hydrogenation of but-3-en-1-ol (**11a**) S42
Figure S27. Conversion vs time plots for hydrogenation of but-3-en-1-ol (**11a)** S43
Figure S28. ^{1}H NMR spectra for homogeneous hydrogenation of pent-2-en-1-ol (**12a**) S44
Figure S29. ^{1}H NMR spectra for heterogeneous hydrogenation of pent-2-en-1-ol (**12a**) S45
Figure S30. Hydrogenation of crotyl alcohol (**13a**) with **2-OTs** and **3** S46
Figure S31. Gravimetric n-butanol uptake S47
Figure S32. Product inhibition test S48
Figure S33. GC product distribution and calibration curves S49
6. References S50

1. Materials, methods and instrumentation

General procedures: All manipulations of air and moisture sensitive compounds were performed under an inert gas atmosphere (N_2 or Ar) using standard Schlenk and cannula techniques or a MBraun glovebox. Glassware was dried in an oven at 130°C prior to use.

Solvents: Pentane and CH_2Cl_2 were dried using a MBraun SPS-800 solvent purification system and stored over 3 Å molecular sieves, pre-activated at 120 °C under vacuum for 20 h. CD_2Cl_2 was dried by stirring over CaH_2 overnight and then it was vacuum-distilled onto pre-activated 3 Å molecular sieves. Anhydrous acetone was bought from Acros Organics and used as received. All above solvents were degassed before use by three freeze-pump-thaw cycles. All other solvents were bought from Fisher (reagent-grade) and used as received.

Reagents: $[IrCl(cod)]_2$[1] and $[IrCl(cod)(PCy_3)]$[2] were prepared by the literature procedures. Crabtree's catalyst $[Ir(cod)(PCy_3)(py)][PF_6]$ (**2-PF$_6$**) was bought from Alfa-Aesar (99%). All Ir complexes were stored in the glovebox and handled under anaerobic conditions. Acetic acid/ sodium acetate buffer solution (pH = 4.7) was bought from Honeywell Fluka. All other chemicals were bought either from Sigma Aldrich or TCI-Chemicals. Alkenes were dried over CaH_2 and olefinic alcohols (> 95% *trans* isomers) were dried over Mg turnings. All substrates were then vacuum-distilled onto pre-activated 3 Å molecular sieves.

Analysis: CHN-S/F elemental analysis was conducted by Mrs Jean Ellis at the University of Liverpool or by Mr Stephan Boyer at London Metropolitan University. ICP-OES analysis (Cr, Na, S, Co, Ir, P) was performed by Mr Steven Moss at the University of Liverpool or Mr Neil Bramall at the University of Sheffield. Samples (5-10 mg) were digested in 10 mL of 0.1 M KOH and then diluted 5-fold with milli-Q H_2O (18 MΩ cm at 25 °C). Thermogravimetric analysis (TGA) was carried out using a Q500 TA instrument in the 25-750 ºC temperature range with a 10 ºC/min ramping rate and a 50 mL/min stream of air.

Scanning Electron Microscopy (SEM) imaging was performed with a Hitachi S-4800 Field Emission SEM. A small amount of sample powder was spread on a carbon tape attached to an Al stab and coated with a few nanometers thick layer of gold.

N_2 adsorption-desorption isotherms at 77 K were collected on a Micromeritics Tristar instrument. Samples were outgassed under vacuum at 125 °C for 20 h before measurement. The specific surface area was calculated by applying the BET model for the N_2 adsorption data over the range of p/p_0 = 0.05 – 0.2, selected according to the consistency criteria of Rouquerol. Total pore volume was estimated from the N_2 adsorbed amount at p/p_0 = 0.5. Pore size

distribution was determined by employing the Tarajona NLDFT model,[3] assuming cylindrical pores, as implemented in MicroActive program (Version 4.03) from Micromeritics.

GC analysis was performed with an Agilent Technologies 7890A gas chromatograph equipped with a programmed split/splitless injector and FID, using a DB-WAXetr 60 m × 0.25 mm *i.d.*, 0.25 μm film thickness capillary column (Agilent J&W).

GC Method for alkenes: He carrier gas, injector temperature = 260 °C, pressure = 15 psi, injection volume = 1 μL (split ratio = 90:1), heating rate = 10 min at 40 °C, then ramp to 150 ° at 20 °C/min.

GC Method for olefinic alcohols: He carrier gas, injector temperature = 260 °C, pressure = 25 psi, injection volume = 1 μL (split ratio = 60:1), heating rate = 5 min at 50 °C, then ramp to 120 ° at 5 °C/min and hold for 10 min, then ramp to 220 ° at 20 °C/min and hold for 5 min.

Response factors were determined after calibration with the commercially available compounds. All samples were filtered via a 0.2 μm syringe filter (Acrodisc® GHP) before injection. Table S6 lists all retention times for alkenes and olefinic alcohols.

ESI-MS data were collected on a Bruker MicroTOF interfaced with a glovebox.[4]

2. NMR Spectroscopy

Solution phase ^{1}H, ^{13}C, ^{31}P and ^{19}F NMR spectra were collected on a Bruker Ascend 400 MHz spectrometer at room temperature unless otherwise stated. MOFs were digested in 0.5 M NaOD solution in D_2O. ^{1}H NMR spectra were referenced to residual solvent signals. ^{31}P{^{1}H} NMR spectra were referenced externally to 85% H_3PO4 (D_2O). All chemical shifts (δ) are quoted in ppm and coupling constants in Hz.

Solid state NMR spectra were collected by Dr Nicholas H. Rees (University of Oxford) at 294 K on a Bruker Avance III HD spectrometer equipped with a 9.4 Tesla magnet, operating at 100.6 MHz for ^{13}C, 162 MHz for ^{31}P, 376 MHz for ^{19}F and 61 MHz for ^{2}H using either a 4 mm or a 3.2 mm zirconia rotor containing approximately 70 and 35 mg of sample, respectively, and a MAS rate of 10 – 40 kHz. ^{2}H spectra were acquired using a contact time of 2.5 ms, an acquisition time of 13 ms and a recycle delay of 1 s. ^{31}P{^{1}H} spectra were acquired using a contact time of 2 ms, an acquisition time of 10.7 ms and a recycle delay of 5 s. All ^{2}H spectra were referenced to TSP (trimethylsilyl propionate, 0 ppm). All ^{31}P{^{1}H} spectra were referenced to ADP (ammonium dihydrogen phosphate, 1 ppm).

Gas phase ^{1}H NMR spectroscopy was carried out using a Bruker Ascend 400 MHz spectrometer. The T1 delay was set to 1 s, as this has been previously shown to allow for the accurate comparison of integrals.[5] Samples were loaded into a high-pressure NMR tube sealed with a Teflon stopcock, before being transferred to a Schlenk vacuum line, evacuated and then loaded with the gaseous reagents according to the literature procedure.[6] For catalytic hydrogenation runs, the spectrometer was initially locked and shimmed to a separate C_6D_6 sample in a similar bore tube. The sample was then replaced with the NMR tube containing the catalyst and the gaseous reagents (full experimental details of hydrogenation are provided later).

3. X-ray diffraction (XRD)

Powder XRD patterns were collected in transmission geometry at 298 K on a Bruker D8 advance diffractometer with Cu Kα radiation (λ = 1.54184 Å). Samples were ground and transferred to a 0.7 mm *i.d.* borosilicate capillary. Profiles were collected in the 3º< 2θ < 40º range with a 2θ step size of 0.028º over a 1 h period. Le Bail fits[7] were carried out with the PANanalytical X´Pert HyScore Plus program (version 2.2.1).

Single crystal XRD data for [Ir(cod)(PCy_3)(Py)][OTs] (**2-OTs**) and [Ir(cod)(PCy_3)(OTs)] (**3**) were collected as follows: a suitable crystal was mounted on a MiTeGen Micromounts using perfluoropolyether oil and cooled rapidly to 150 K in a stream of nitrogen gas using an Oxford Cryosystems Cryostream unit.[8] Data were collected with an Agilent SuperNova diffractometer (Cu Kα radiation, λ = 1.54184 Å). Raw frame data were reduced using CrysAlisPro.[9] The structure was solved using SHELXT[10] and refined using full-matrix least squares refinement on all F^2 data using SHELXL-14[11] with OLEX2 program.[12] All hydrogen atoms were placed in calculated positions (riding model). Crystallographic data have been deposited to the Cambridge Crystallographic Data Centre under CCDC 1573593 (**2-OTs**) and 1573594 (**3**). Crystal structures of **2-OTs** and **3** are shown in Figures S16 and S17, respectively. Most important bond lengths and angles are listed in Table S4 and crystallographic details are provided in Table S5. These data can be obtained free of charge from the Cambridge Crystallographic Data Centre via www.ccdc.cam.ac.uk/data_request/cif

4. Synthesis

1-SO_3H: $[H_{1.8(1)}Na_{0.2(1)}][Cr_3(\mu_3\text{-}O)(BDC\text{-}SO_3)_3].46(H_2O)$

1-SO_3H is a robust and mesoporous anionic MOF, readily synthesized on a multi-gram scale using cheap commercially available chemicals. The reported procedure[13] was slightly modified. A CrO_3 stock solution was initially prepared by dissolving 3.23 g (= 32.3 mmol) in 50 mL H_2O ([Cr] = 0.646 M). Subsequently, 2.00 g (= 7.46 mmol) of the $C_8H_3SO_7Na$ linker (**H_2BDC-SO_3Na**) were combined with 11.55 mL (= 7.46 mmol) of the CrO_3 stock solution in a 40 ml glass vial. Concentrated HCl 37 % was added (1.5 equivalent, 934 µL = 11.18 mmol) and the suspension was diluted with H_2O to a total volume of 30 mL (*in our hands, adding two equivalents of HCl, as reported,*[13-14] *results in very low yields*). The reaction mixture was rapidly stirred for 1 h during which the linker was completely dissolved ([H_2BDC-SO_3Na] = 0.249 M). The resulting solution was then transferred into a 50 mL Parr autoclave. The autoclave was placed inside an oven, heated at 190 °C (2.0 °C/min), maintained at 190 °C for 6 days and then cooled down to room temperature (0.4 °C/min). A forest-green solid was formed which was collected by filtration (Whatman No1 Filter paper), washed with H_2O (600 mL) and MeOH (300 mL) and dried under vacuum. 1.91 g of a pale green powder were isolated: yield is 44% for $C_{24}H_{102.8}Cr_3Na_{0.2}O_{68}S_3$ (FW = 1736.6).

1-SO_3H was thoroughly characterized by ICP-OES and CHN-S elemental analysis (Table S1), PXRD (Le Bail fit is shown in Figure S4), TGA (Figure S8), N_2 adsorption-desorption at 77 K (Figure S9) and SEM imaging (Figure S11). ICP-OES and CHN-S elemental analysis of five different samples gave an overall formula of $[H_{1.8(1)}Na_{0.2(1)}][Cr_3(\mu_3\text{-}O)(BDC\text{-}SO_3)_3].46(H_2O)$.

1-SO_3Na: $[H_{0.2(1)}Na_{1.8(1)}][Cr_3(\mu_3\text{-}O)(BDC\text{-}SO_3)_3].54(H_2O)$

1-SO_3H (225 mg, 0.13 mmol) was combined with 25 mL of an AcONa/AcOH buffer solution (pH = 4.7) inside a 40 mL vial. The suspension was rapidly stirred, centrifuged and the supernatant was decanted. This process was repeated three times within 24 h. The final product was collected by filtration (Whatman No1 Filter paper), washed with milli-Q H_2O (400 mL) and MeOH (200 mL) and dried under vacuum. 155 mg of a pale green powder were isolated; yield is 62% for $C_{24}H_{117.2}Cr_3Na_{1.8}O_{76}S_3$ (FW = 1915.9).

1-SO_3Na was thoroughly characterized by ICP-OES and CHN-S elemental analysis (Table S1), PXRD (Le Bail fit is shown in Figure S5), TGA (Figure S8), N_2 adsorption-desorption at 77K (Figure S9) and SEM imaging (Figure S11). ICP-OES and CHN-S elemental analysis of five different samples gave a formula of $[H_{0.2(1)}Na_{1.8(1)}][Cr_3(\mu_3\text{-}O)(BDC\text{-}SO_3)_3].54(H_2O)$.

$[Cp^*_2Co]^+$@1-SO_3Na: $[(Cp^*_2Co)_{0.3}Na_{1.7}][Cr_3(\mu_3\text{-}O)(BDC\text{-}SO_3)_3].46(H_2O)$

Synthesis was carried out under air. A $[Cp^*_2Co][PF_6]$ stock solution was initially prepared by dissolving 1.12 g (= 2.36 mmol) in 50 mL acetone ($[Cp^*_2Co]$ = 0.047 M). **1-SO_3Na** (60 mg, 0.031 mmol) was combined with 1.28 mL (= 0.060 mmol) of the $[Cp^*_2Co][PF_6]$ stock solution in a 40 ml glass vial and the suspension was diluted with acetone to a total volume of 3.0 mL to set nominal $[Cp^*_2Co]^+$ = 0.020 M. The suspension was stirred for 24 or 48 h. A pale green powder was collected by filtration, washed thoroughly with acetone until the filtrate was colourless (5 x 20 mL) and dried under vacuum. 50 mg were isolated: yield is 89% for $C_{30}H_{110}Co_{0.3}Cr_3Na_{1.7}O_{68}S_3$ (FW = 1868.1).

$[Cp^*_2Co]^+$@1-SO_3Na was thoroughly characterized by ICP-OES and CHN-S elemental analysis (Table S2), PXRD (Le Bail fit is shown in Figure S6), N_2 adsorption-desorption at 77K (Figure S9), ^{1}H NMR spectroscopy after digestion in 0.5 NaOD (Figure S10) and SEM imaging (Figure S12). Elemental analysis suggested that cation exchange practically stopped after one day and approximately 15% of the entrapped cations were exchanged with $[Cp^*_2Co]^+$. This was verified by ^{1}H NMR spectroscopy in solution after digestion (Figure S10).

2@1-SO_3Na: Cation exchange of Crabtree's catalyst (**2-PF_6**) in **1-SO_3Na**

Synthesis was carried out under strictly anaerobic conditions. **1-SO_3Na** was activated under vacuum (10^{-3} mbar) at 125°C overnight, transferred into the glovebox and added into a 50 mL J-Young tube (60 mgs, 0.064 mmol of desolvated **1-SO_3Na**, FW = 943.1). **2-PF_6** (80 mg, 0.10 mmol) was dissolved in 5 ml of dry and degassed acetone ([**2-PF_6**] = 0.020 M) and then cannula-transferred into the J-Young tube containing the desolvated **1-SO_3Na** MOF. The J-Young tube was sealed and gently shaken for 20 h after which the supernatant was filtered off using a filter-cannula. The pale green powder was thoroughly washed with dry acetone until the washings were colourless (4 x 5 mL) and then soaked in dry acetone for 20 h. Subsequently, the supernatant was filtered off as before and the material was dried under vacuum until the particles could move freely.

Characterization of **2@1-SO_3Na** was carried out with the aid of ICP-OES (Table S3), PXRD (Le Bail fit is shown in Figure S7), N_2 adsorption-desorption at 77K (Figure S9), NMR spectroscopy (Figure 2c, Figures S14-S15) and SEM imaging (Figure S12). All analytical data concur that **2** is encapsulated intact inside the pores of **1-SO_3Na**, replacing 7% of the charge balancing cations.

[Ir(cod)(py)$_2$][OTs]

[IrCl(cod)]$_2$ (338 mg, 0.503 mmol), Ag[OTs] (287 mg, 1.03 mmol) and excess pyridine (0.1 mL) were added to a Schlenk flask. CH_2Cl_2 (25 mL) was then added and the resulting mixture was stirred for 16 h at ambient temperature in the absence of light. Solids were allowed to settle and the solution was then collected by filtration. The solvent was removed under vacuum and the resulting sticky yellow solid was triturated with pentane (2 × 20 mL). The resulting yellow powder was then dried under vacuum. 512 mg were isolated (81% yield).

Elemental analysis (calculated values in parenthesis) for $C_{25}H_{29}IrN_2O_3S{\cdot}½CH_2Cl_2$ (FW = 672.26): C 46.00 (45.56), H 4.62 (4.50), N 4.00 (4.17)

^{1}H NMR (CD_2Cl_2, 400 MHz): δ_H = 8.74 (m, 4H, ortho-H Py), 7.76 (m, 2H, para-H Py), 7.68 (d, 2H, J_{HH} = 8 Hz, OTs), 7.47 (m, 4H, meta-H Py), 7.13 (d, 2H, J_{HH} = 7.8 Hz, OTs), 3.86 (br s, 4H, alkene CH), 2.42 (br, 4H, CH_2), 2.34 (s, 3H, CH_3), 1.75 (m, 4H, CH_2).

ESI-MS (1,2-$C_6H_4F_2$, 60°C, 4.5 kV): m/z 459.14 (calculated 459.14 for $[Ir(COD)(Py)_2]^+$ fragment).

2-OTs: [Ir(cod)(PCy$_3$)(py)][OTs]

A bright yellow solution of [Ir(cod)(Py)$_2$][OTs] (64 mg, 0.095 mmol) in CH_2Cl_2 (10 mL) was treated dropwise with a colorless solution of PCy_3 (27.5 mg, 0.095 mmol). An immediate color change to red was observed. After stirring for 1 h at ambient temperature the solution was isolated by filtration. The solvent was then removed under vacuum and the resulting sticky red solid was triturated with pentane (2 × 10 mL). The resulting red powder was then thoroughly dried under vacuum. 50 mg were isolated (63% yield).

Elemental analysis (calculated values in parenthesis) for $C_{38}H_{57}IrNO_3PS$ (FW = 831.1): C 54.91 (54.92), H 6.97 (6.91), N 1.77 (1.69)

^{1}H and ^{31}P{^{1}H} solution NMR spectra of **2-OTs** in CD_2Cl_2 exhibited broad peaks at 298 K, due to a reversible ligand exchange process between pyridine and tosylate producing a mixture of **2-OTs** and **3**. This process practically stopped at 193 K with a **2-OTs**/**3** ratio equal to 9/1.

^{1}H NMR (CD_2Cl_2, 400 MHz, 193 K, selected resonances): δ_H 8.63 (m, 2H, ortho-H Py), 7.93 (m, 1H, para-H Py), 7.63-7.57 (m, 4H, overlapping OTs and meta-H Py), 7.11 (d, 2H, J_{HH} = 7.9 Hz, OTs), 3.92 (br s, 2H, alkene CH), 3.84 (br s, 2H, alkene CH), 2.33-2.09 (m, 7H, overlapping COD CH_2 and CH_3), 1.90-0.91 (m, 37H, overlapping aliphatic CH).

^{31}P{^{1}H} NMR (CD_2Cl_2, 162 MHz, 193 K): δ_P = 9.5 (s).

^{1}H and ^{31}P{^{1}H} solution NMR spectra of **2-OTs** were also measured at 298 K in the presence of one excess equivalent of pyridine. Exchange between the free and coordinated pyridine was

noted at this temperature, with OTs solely acting as the counter anion. Figures S18-S20 show related ^{1}H, $^{31}P\{^{1}H\}$ and EXSY NMR spectra, revealing a ligand exchange between pyridine and tosylate.

^{1}H NMR (CD_2Cl_2, 400 MHz, 298 K, selected resonances): δ_H = 8.71 (br s, 2H, ortho-H Py), 7.97 (br s, 1H, para-H Py), 7.74-7.60 (br m, 4H, overlapping OTs and meta-H Py), 7.12 (d, J_{HH} 7.7 Hz, 2H, OTs), 4.09 (br s, 2H, alkene CH), 3.93 (br s, 2H, alkene CH), 2.41-2.19 (m, 6H, overlapping aliphatic CH), 2.01-1.67 (m, 22H, overlapping aliphatic CH), 1.59-1.45 (m, 6H, overlapping aliphatic CH), 1.35-1.22 (m, 4H, overlapping aliphatic CH), 1.14-1.00 (m, 6H, overlapping aliphatic CH).

$^{31}P\{^{1}H\}$ NMR (CD_2Cl_2, 162 MHz, 298 K): δ_P = 10.4 (s).

$^{31}P\{^{1}H\}$ MAS NMR (spinning rate of 10 kHz): δ_P = 5.6 (s)

ESI-MS (1,2-$C_6H_4F_2$, 60°C, 4.5 kV): m/z 660.33 (calculated 660.33 for $[Ir(COD)(Py)(PCy_3)]^+$ fragment).

[Ir(cod)(PCy$_3$)(OTs)] (3):

A stirred slurry of Ag[OTs] (124 mg, 0.44 mmol) in CH_2Cl_2 (15 mL) was combined with a solution of [IrCl(cod)(PCy$_3$)] (184 mg, 0.30 mmol) in CH_2Cl_2 (20 mL). The resulting mixture was stirred for 16 h at ambient temperature in the absence of light. Solids were allowed to settle and the solution was then isolated by filtration. The solvent was then removed under vacuum and the resulting yellow powder was washed with pentane (2 × 10 mL). The powder was then dried under vacuum. 165 mg were isolated (73% yield).

Elemental analysis (calculated values in parenthesis) for $C_{33}H_{52}IrO_3PS$ (FW = 752.0): C 52.65 (52.71), H 6.94 (6.97).

^{1}H NMR (CD_2Cl_2, 400 MHz): δ_H = 7.68 (d, 2H, J_{HH} = 8 Hz, OTs), 7.21 (br d, 2H, J_{HH} = 7.6 Hz, OTs), 5.53 (br s, 2H, alkene CH), 3.18 (br s, 2H, alkene CH), 2.36 (s, 3H, CH_3), 2.23-2.12 (m, 4H, COD CH_2), 1.92-1.62 (m, 20H, overlapping aliphatic CH), 1.59-1.46 (m, 8H, overlapping aliphatic CH), 1,30-1.19 (m, 4H, overlapping aliphatic CH), 1.13-1.00 (m, 6H, overlapping aliphatic CH). Addition of pyridine in a CD_2Cl_2 solution of **3** leads to **2-OTs**.

$^{31}P\{^{1}H\}$ NMR (CD_2Cl_2, 162 MHz): δ_P = 17.8 (s).

$^{31}P\{^{1}H\}$ MAS NMR (spinning rate of 10 kHz): δ_P = 13.7 (s)

5. Catalysis

Heterogeneous hydrogenation of alkenes with 2@1-SO_3Na:

Alkenes were purified as described in materials, methods and instrumentation. Stock solutions were prepared for each substrate in CH_2Cl_2. Reactions were carried out in J-Young tubes. For hydrogenations at 1000 ppm loading (0.1 mol %), a 50 mL tube was used. For lower loadings, larger J-Young tubes were used with a volume of 100 mL (100 ppm loading) or 300 mL (< 100 ppm loading) in order to increase the amount of available H_2. The following procedure describes hydrogenation of 1-octene (**4**) at 1000 ppm loading. Nominal concentrations and amounts of reagents were adjusted accordingly for other substrates and loadings (see Table 1 in the manuscript).

A stock solution of 1-octene was prepared by dissolving 500 μL (= 3.186 mmol) in 2.6 mL of dry and degassed CH_2Cl_2 ([1-octene] = 1.0 M). In the glovebox, 4 mg of **2@1-SO_3Na** (2.28 wt % Ir, 91.2 μg Ir, 0.474 μmols Ir) were added into a 50 mL J-Young tube. The J-Young tube was transferred to a Schlenk line, outgassed for 5 min and backfilled with N_2. 500 μL of the 1-octene stock solution (= 0.5 mmol) were added anaerobically via a 1 mL syringe and the suspension was diluted with 500 μL of dry and degassed CH_2Cl_2 (V_{total} = 1.0 mL, [1-octene] = 0.5 M). The J-Young tube was transferred to the hydrogenation line, immersed in liquid N_2 for 10 min and then the overhead space was evacuated. The tube was backfilled with H_2 while still fully immersed in liquid N_2 (P_{H2} = 1 bar at 77 K) and then placed in a water bath at 20 °C (P_{H2} = 3.8 bar at 293 K, 7.8 mmol of H_2 for a 50 mL J-Young tube). The suspension was stirred for 3 h at 300 rpm after which the tube was depressurized by immersion in liquid N_2 for 10 min and slow evacuation of the overhead space for 1 minute to remove any H_2 excess. Finally, the tube was left to thaw back to ambient temperature and backfilled with N_2.

The product distribution was determined by GC (refer to general methods for more details). The supernatant was filtered via a syringe filter (0.2 μm, Acrodisc® GHP) and then 200 μL were diluted to 1 mL with CH_2Cl_2. Retention times are provided in Table S6. Repeated reactions delivered a product distribution that was reproducible to within ± 5 %.

Leaching test: A heterogeneous hydrogenation reaction was set up with **2@1-SO_3Na** as the catalyst and 1-octene as the substrate (100 ppm loading, [1-octene] = 1.0 M, V = 4.0 mL). After 3 h, the supernatant was filtered off via a filter-cannula and the filtrate was collected in a J-Young tube and transferred inside a N_2 purged glove-bag. The supernatant was filtered once more in the glove bag using a 0.2 μm syringe filter (Acrodisc® GHP) and subsequently an

aliquot of 100 μL was diluted to 1 mL with CH_2Cl_2 and analyzed by GC (35% conversion exclusively to *n*-octane, TON = 3500). The remaining filtrate was then re-pressurized with H_2 and left to react overnight. Aliquots (100 μL) were collected after 3 h and 21 h and analyzed by GC. No more conversion was observed, revealing that turnover stopped once the heterogeneous catalyst was separated from the reaction mixture (Figure S21).

Recycling experiments: A heterogeneous hydrogenation reaction was set up with **2@1-SO_3Na** as the catalyst and 1-octene as the substrate (100 ppm loading, [1-octene] = 1.0 M, V = 4.0 mL). After the end of the first cycle (20 h), the supernatant was filtered off using a filter cannula and analyzed by GC (100% conversion to n-octane). Subsequently, a fresh batch of 1-octene in CH_2Cl_2 was added ([1-octene] = 1.0 M) and hydrogenation was carried out as before (97% conversion selectively to *n*-octane for the 2nd cycle). The whole process was repeated once more. Conversion for the 3rd cycle was 82% (Figure S22).

Heterogeneous hydrogenation of olefinic alcohols with 2@1-SO_3Na:

Olefinic alcohols were purified as described in materials, methods and instrumentation. The *trans* isomers (>95%) of **12a** and **13a** were used. Stock solutions were prepared for each substrate in CH_2Cl_2. Reactions were carried out at 1000 ppm loading (0.1 mol %) in 50 mL J-Young tubes. The following procedure describes hydrogenation of but-3-en-1-ol (homoallylic alcohol **11a**). Amounts of reagents were adjusted accordingly for other olefinic alcohols.

A stock solution of **11a** was prepared by dissolving 0.400 mL (= 4.65 mmol) of the purified substrate in 4.1 mL of dry and degassed CH_2Cl_2 ([**11a**] = 1.03 M). In the glovebox, 3 mgs of **2@1-SO_3Na** (2.28 wt % Ir, 68.4 μg Ir, 0.356 μmols Ir) were added into a 50 mL J-Young tube. The J-Young tube was mounted on the Schlenk line, outgassed for 5 min and backfilled with N_2. 340 μL of the **11a** stock solution (= 0.350 mmol) were added anaerobically via a 1 mL syringe and the suspension was diluted with 360 μL of dry and degassed CH_2Cl_2 (V_{total} = 0.70 mL, [**11a**] = 0.50 M). The J-Young tube was transferred to the hydrogenation line, immersed in liquid N_2 for 10 min until the reaction solution was frozen and then the overhead space was evacuated. The tube was backfilled with H_2 while still fully immersed in liquid N_2 (P_{H2} = 1 bar at 77 K) and then placed in a water bath at 20 °C (P_{H2} = 3.8 bar at 293 K, 7.8 mmol of H_2 for a 50 mL J-Young tube). The suspension was stirred for 20 h at 300 rpm after which the tube was depressurized by immersion in liquid N_2 for 10 min and slow evacuation of the overhead space for 1 minute to remove any H_2 excess. Finally, the tube was left to thaw back to ambient temperature and backfilled with N_2.

Product distribution was determined by ^{1}H NMR. The reaction mixture was filtered using a 0.2 μm syringe filter (Acrodisc® GHP) and subsequently an aliquot of 200 μL was diluted to 1 mL with a 0.071 M mesitylene solution, used as standard, in $CDCl_3$. Product distribution and mass-balance was determined by integration of the peaks of the aromatic protons of mesitylene and the characteristic peaks of the substrate and the products (Figures S24-S26, S28-S29). According to NMR analysis, all reactions were mass-balanced within ± 7%. For homoallylic alcohol **11a**, product distribution and conversion was also determined by GC (Table S6 and Figure S33). Results lie in very good agreement with NMR and reaction is mass-balanced within ± 5%.

Homogeneous hydrogenation with Crabtree's catalyst:

Homogeneous reactions were always run "side by side" with their heterogeneous counterparts. A stock solution of **2-PF$_6$** was initially prepared inside a J-young tube by dissolving 3.5 mg (= 4.35 μmol) in 4.2 mL of dry and degassed CH_2Cl_2 ([**2-PF$_6$**] = 1.04 x 10^{-3} M). Homogeneous hydrogenation was then carried out as described above by combining the required amounts of the two stock solutions (**2-PF$_6$** and substrate) and diluting if necessary with CH_2Cl_2 for the substrate concentration and loading to be identical with these of the corresponding heterogeneous reaction. When alkenes were employed as substrates, the reaction mixture was filtered through a silica pad (40 – 63 μm particle size) after the end of the reaction. The pad was thoroughly washed with CH_2Cl_2 (2 x 20 mL). The filtrate was diluted to a total volume of 50 mL and analyzed by GC. Table S6 lists the retention times. When olefinic alcohols were employed as substrates, product distribution was determined by ^{1}H NMR, as described above for the heterogeneous system.

Selective poisoning experiments:

Table S7 shows conversions and selectivities for the following experiments:

a) Homogeneous hydrogenation of crotyl alcohol (**13a**, 95% *trans* isomer) with **2-PF$_6$** as the catalyst at 0.1 mol % loading was carried out in two 50 mL J-Young tubes as above, but at a slightly higher substrate concentration ([**13a**] = 0.75 M). Before addition of hydrogen, approximately 180 equivalents of freshly distilled and degassed butanal (**13d**) were added in one of the tubes. Both tubes were then pressurized with H_2. After 3h, H_2 was removed as previously described and the relative concentration of all the compounds in each tube was determined by ^{1}H NMR spectroscopy. Addition of butanal had a minor effect on final product distribution, suggesting that it does not poison the catalyst (Figure S32a). The same experiment

was performed with the heterogeneous catalyst **2@1-SO_3Na**. Addition of butanal also had a negligible effect on final product distribution (Figure S32b).

b) In a separate experiment, a homogeneous hydrogenation of crotyl alcohol (**13a**) with **2-PF_6** as the catalyst at 0.1 mol % loading was carried out in three 50 mL J-Young tubes ([**13a**] = 0.5 M). After 3 h, H_2 was removed as previously described and the reaction mixture of the 1st tube was directly analyzed with ^{1}H NMR spectroscopy (54% conversion). In the 2nd tube, a fresh batch of the homogeneous catalyst **2-PF_6** was added, whereas the contents of the 3rd tube were cannula-transferred over to a J-Young tube containing the heterogeneous catalyst **2@1-SO_3Na**. A fresh batch of H_2 was added in both tubes and stirring was applied for 20 h. Complete conversion was observed for both experiments, indicating that the reaction mixture does not poison either catalyst.

c) A final test was carried out in which the homogeneous system was re-pressurized with H_2 after 3 h and left to react for 20 h. Conversion did not significantly increase, suggesting that the homogeneous catalyst was deactivated after 3 h.

Gas phase hydrogenation of 1-butene:

Gas/solid hydrogenation of 1-butene was carried out according to the literature procedure.[6] A high pressure NMR tube (volume ≈ 1.8 mL) was loaded with finely powdered catalyst (~0.5 mg) inside a glovebox. Subsequently, the high pressure NMR tube was transferred to a schlenk line and the argon atmosphere was replaced with 1-butene (1 atm). The tube was sealed and frozen in liquid nitrogen, hydrogen (1 atm) was introduced at 77 K. As a timer was simultaneously started, the tube was quickly sealed, thawed and transferred to a NMR spectrometer. Experimental results (TONs, TOFs and recycling) are shown in Figure S23.

Gas phase NMR were calibrated to H_2 (δ = 4.50 ppm).

Gas phase chemical shifts of butenes and butane are as follows:

1-Butene: δ 5.87 (m, 1H, C*H*CH_2), 4.99 (d, J = 17.0 Hz, 1H, Z-C*H*), 4.88 (d, J = 9.7 Hz, 1H, E-C*H*), 2.09 (m, 2H, CH_2CH_3 (C*H*$_2$)), 1.04 (t, J = 7.4 Hz, 3H, CH_2C*H*$_3$).

2-Butene (mixture of *E* and *Z*-isomers): δ 5.48 (br s, C*H*), 1.61 (br s, C*H*$_3$).

Butane: δ 1.38 (br s, 4H, C*H*$_2$$CH_3$), 0.97 (br s, 6H, CH_2C*H*$_3$).

Table S1. ICP-OES and CHN-S elemental analysis for **1-SO$_3$H** and **1-SO$_3$Na**.

**1-SO$_3$H**	$[H_{1.8(1)}Na_{0.2(1)}][Cr_3(\mu_3\text{-}O)(BDC\text{-}SO_3)_3].46(H_2O)$ $C_{24}H_{102.8}Cr_3Na_{0.2}O_{68}S_3$ (FW = 1736.6)	
Element	Found (%)	Calculated (%)
Cr[a]	9.37	8.98
S[b]	5.30	5.54
C[b]	16.05	16.60
H[b]	5.35	5.97
Na[a]	0.19	0.26
H_2O[c]	45.8	47.7
**1-SO$_3$Na**	$[H_{0.2(1)}Na_{1.8(1)}][Cr_3(\mu_3\text{-}O)(BDC\text{-}SO_3)_3].54(H_2O)$ $C_{24}H_{117.2}Cr_3Na_{1.8}O_{76}S_3$ (FW = 1915.9)	
Element	Found (%)	Calculated (%)
Cr[a]	8.18	8.14
S[b]	4.69	5.02
C[b]	14.59	15.05
H[b]	5.78	6.17
Na[a]	2.21	2.16
H_2O[c]	50.7	50.8

[a] ICP-OES analysis after digesting 10 mgs in 10 mL 0.1 M KOH and then diluting 5-fold with milli-Q H_2O; [b] CHN-S elemental analysis; [c] TGA analysis (Figure S8)

Table S2. ICP-OES and CHN-S elemental analysis for **$[Cp^*_2Co]^+$@1-SO_3Na**.

Formula	$[(Cp^*_2Co)_{0.3}Na_{1.7}][Cr_3(\mu_3\text{-}O)(BDC\text{-}SO_3)_3].46(H_2O)$			
	$C_{30}H_{110}Cr_3Co_{0.3}Na_{1.7}O_{68}S_3$ (FW = 1868.1)			
Time	Cation exchange for 1 day		Cation exchange for 2 days	
Element	Found (%)	Calculated (%)	Found (%)	Calculated (%)
Cr[a]	8.09	8.35	8.17	8.35
S[b]	4.65	5.15	4.67	5.15
C[b]	19.15	19.29	19.16	19.29
H[b]	5.41	5.94	5.74	5.94
Na[a]	1.92	2.09	1.90	2.09
Co[a]	1.07	0.95	1.10	0.95
H_2O[c]	43.5	44.4	42.5	44.4

Conditions: 0.020 M solution of $[Cp^*_2Co][PF_6]$ in acetone, 2/1 initial $[Cp^*_2Co]^+$/MOF ratio. [a] ICP-OES analysis after digesting 10 mgs in 10 mL 0.1 M KOH and then diluting 5-fold with milli-Q H_2O; [b] CHN-S elemental analysis; [c] % weight loss after activation at 125 °C for 20 h.

Table S3. ICP-OES[a] analysis for **2@1-SO_3Na**.

	$[H_{0.2(1)}Na_{1.65(10)}\{Ir(cod)(PCy_3)(py)\}_{0.15(5)}][Cr_3(\mu_3\text{-}O)(BDC\text{-}SO_3)_3].(Me_2CO)_{3\text{-}5}$[b]					
	Sample A (with 5 Me_2CO molecules)			Sample B (with 3 Me_2CO molecules)		
Element	Found (%)	Calculated (%)	Atomic[c]	Found (%)	Calculated (%)	Atomic[c]
Cr	11.98	11.74	3.00(5)	12.64	12.86	3.00(5)
S	7.80	7.24	3.00[c]	8.43	7.93	3.00[c]
Na	2.25	2.85	1.65(10)	2.57	3.13	1.65(10)
Ir	2.27	2.17	0.15(5)	2.28	2.38	0.15(5)
P	0.35	0.35	0.15(5)	0.36	0.38	0.15(5)

[a] Digestion of 5 mgs in 10 mL 0.1 M KOH and then diluting 5-fold with milli-Q H_2O; [b] Solvent content different per sample; [c] Atomic ratios normalized to 3 sulfur atoms, experimental error in parenthesis.

Table S4. Selected bond lengths (Å) and angles (°) for complexes **2-PF$_6$**[15], **2-OTs**[a] and **3**.[a]

Compound[b]	**2-PF$_6$**	**2-OTs**	**3**
Ir1-P1	2.368(2)	2.3378(6)	2.3391(9)
Ir1-N1/O1	2.088(3)	2.105(2)	2.105(2)
Ir1-C1	2.146(3)	2.135(3)	2.105(3)
Ir1-C2	2.163(3)	2.148(3)	2.096(3)
Ir1-C5 (*trans* P)	2.176(3)	2.183(2)	2.226(3)
Ir1-C6 (*trans* P)	2.195(3)	2.235(2)	2.198(3)
C1-C2	1.407(5)	1.405(4)	1.429(6)
C5-C6 (*trans* P)	1.404(5)	1.390(4)	1.390(6)
Ir1-(centroid C1,C2)	2.036	2.023	1.975
Ir1-(centroid C5,C6)	2.070	2.097	2.100
P1-Ir1-N1/O1	92.17(9)	94.15(6)	83.95(7)
(centroid C1,C2)-Ir1-(centroid C5,C6)	86.37	86.38	86.50
(centroid C1,C2)-Ir1-N1/O1	171.31	168.81	177.64
(centroid C5,C6)-Ir1-P1	178.28	165.89	173.72

[a] This work. [b] Single crystal structures are presented in Figure S3 for **2-PF$_6$**, Figure S16 for **2-OTs** and Figure S17 for **3**.

Table S5. Crystallographic details.

	**2-OTs**	**3**
Formula	$C_{38}H_{57}IrNO_3PS$	$C_{33}H_{52}IrO_3PS$
M_r / g mol^{-1}	831.07	751.97
CCDC number	1573593	1573594
Crystal System	Triclinic	Monoclinic
Space Group	P$\bar{1}$ (no. 2)	P2$_1$/n (no. 14)
a / Å	9.5875(3)	10.65960(10)
b / Å	12.9482(4)	11.80880(10)
c / Å	14.8006(5)	25.5623(4)
α / °	98.516(3)	90
β / °	100.274(3)	98.0560(10)
γ / °	100.083(3)	90
V / Å^3	1749.22(10)	3185.95(6)
Z	2	4
D_c / g cm^{-3}	1.578	1.568
T [K]	150.01(10)	150.01(10)
Color and shape	orange prism	yellow-orange block
Size /mm	0.09 × 0.03 × 0.02	0.06 × 0.03 × 0.02
λ / Å	1.54184	1.54184
μ / mm^{-1}	8.665	9.437
2θ range	8.476 – 152.308	6.986 – 152.446
F(000)	848.0	1528.0
Reflections collected	16904	37772
Unique	7221	6630
R_{int}	0.0327	0.0473
Absorption correction	multi-scan	multi-scan
transmission max/min	1.000/0.912	1.000/0.712
unique data [$F_o > 2\sigma F_o$)]	6587	5715
parameters/restraints	407/0	353/0
Goof	1.037	1.023
R_1 [$I > 2\sigma (I)$]	0.0236	0.0334
wR_2 [all data]	0.0518	0.0909
weighting scheme [w]	$1/[\sigma^2(F_o^2) + (0.0191P)^2]$[a]	$1/[\sigma^2(F_o^2) + (0.0579P)^2 + 2.4708P]$ [a]
largest residuals [e Å^{-3}]	0.90, -0.72	2.51, -1.02

[a] $P = 1/3[\max(F_o^2,0) + 2F_c^2]$

Table S6. GC retention times (t_r) of alkenes[a] and alcohols.[b]

Compound	octane	1-octene	cyclohexane	cyclohexene
t_r (min)	8.25	8.95	7.38	8.20
Compound	2-methylhexane	2-methyl-1-hexene	3-methylhexane	3-methyl-1-hexene
t_r (min)	7.03	7.48	7.08	7.16
Compound	Butanal	Butanol	Homallylic	Crotyl (trans & cis)
t_r (min)	6.61	14.12	15.07	16.7 & 17.6

[a] DB-WAXetr 60 m × 0.25 mm *i.d.*, 0.25 µm film thickness capillary column. Injector temperature = 260 °C, pressure = 15 psi; injection volume = 1 µL; split ratio = 90:1; heating rate : 10 min at 40 °C, then ramp to 150 ° at 20 °C/min;. [b] Injector temperature = 260 °C, pressure = 25 psi; injection volume = 1 µL; split ratio = 60:1; heating rate: 5 min at 50 °C, then ramp to 120 °C at 5 °C/min (hold for 10 min), then ramp to 220 °C at 20 °C/min (hold for 5 min).

Table S7. Selective poisoning experiments.[a]

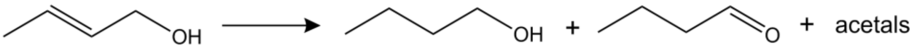

13a **13b** **13d**

Homogeneous hydrogenation with **2-PF₆** (0.1 mol % loading, [crotyl] = 0.75 M)								
	No aldehyde added				180 eqs of aldehyde added[b]			
		Selectivity (%)				Selectivity (%)		
Time	Conv	*n*-butanol	butanal	acetals	Conv	*n*-butanol	butanal	acetals
3 h	69%	36	44	20	76%	35	45	20
Heterogeneous hydrogenation with **2@1-SO₃Na** (0.1 mol % loading, [crotyl] = 0.75 M)								
	No aldehyde added				180 eqs of aldehyde added[b]			
		Selectivity (%)				Selectivity (%)		
Time	Conv	*n*-butanol	butanal	acetals	Conv	*n*-butanol	butanal	acetals
40 h	99%	74	10	16	90%	67	14	19
Catalyst recharging (0.1 mol % loading, [crotyl] = 0.50 M)								
	Recharge with **2-PF₆**[c]				Transfer over to **2@1-SO₃Na**[d]			
		Selectivity (%)				Selectivity (%)		
Time	Conv	*n*-butanol	butanal	acetals	Conv	*n*-butanol	butanal	acetals
20 h	100	28	24	48	>99	>35	27	37

[a] Experimental details are provided on pages S13, S14. [b] Addition of ~180 equivalents of butanal at the beginning of the reaction. [c] Addition of a fresh batch of **2-PF₆** after 3 h. [d] Cannula-transfer of the homogeneous reaction mixture after deactivation (3 h, 54% conversion) over to the heterogeneous catalyst.

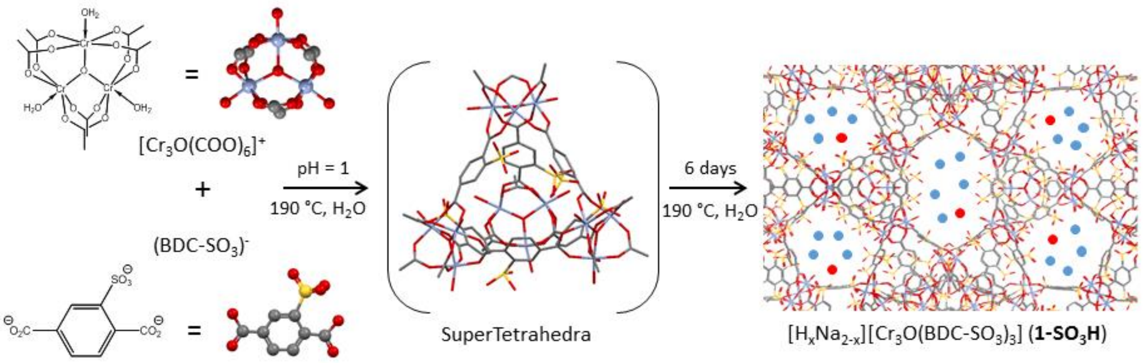

Figure S1. Synthesis of **1-SO$_3$H**.[13] Interconnection of [Cr$_3$(μ_3-O)(O$_2$CR)$_6$]$^+$ nodes with ditopic BDC-SO$_3$ linkers affords microporous super-tetrahedra. Corner-sharing leads to a framework of **mtn** topology with 8 larger (d ~ 3.5 nm) and 16 smaller (d ~ 2.8 nm) mesopores. Each linker is shared between two pores. There are 20 cations per small and 28 cations per large mesopore on average with a total of 20 x 16 + 28 x 8 = 544 cations per unit cell.

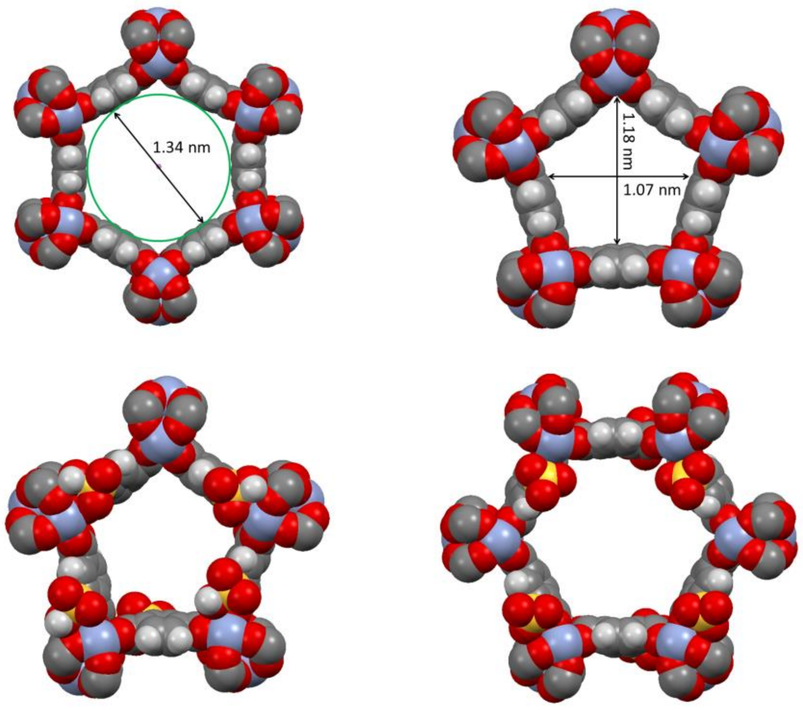

a)

b)

Figure S2. a) In pristine MIL-101(Cr),[16] each unit cell contains two types of mesopores with internal diameters of 2.8 and 3.5 nm, accessible through pentagonal (left) and hexagonal (right) windows with openings of ~1.1 nm and 1.3 nm, after correcting for van der Waals radii.[17] b) Model for the isostructural sulfonated analogue **1-SO$_3$H**. Dimensions of both windows do not change since the sulfonate groups are pointing towards the interior of the pores.

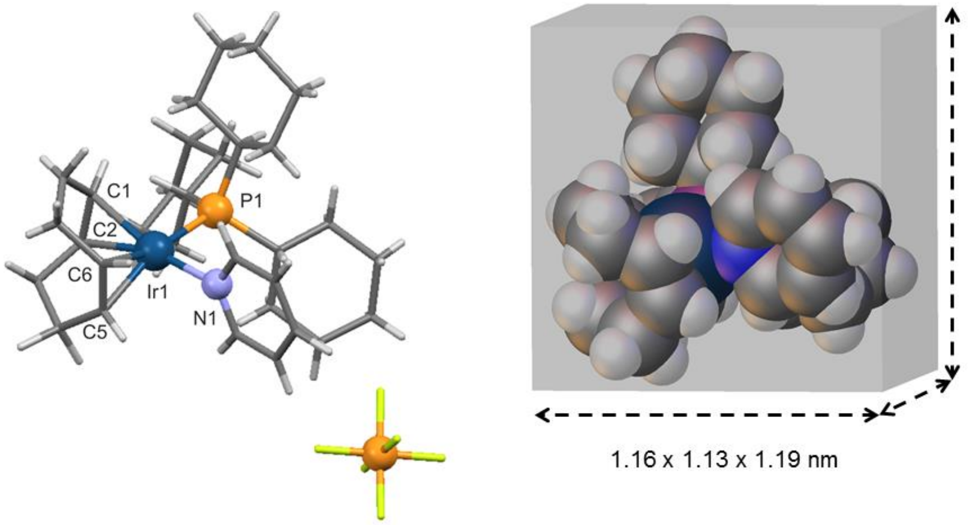

Figure S3. Single crystal structure of **2-PF$_6$** (Crabtree's catalyst)[15] and dimensions of the cation **2** (1.16 x 1.13 x 1.19 nm, V = 1.56 nm^3) by enclosure in a rectangular box, defined by tangents to its van der Waals surface (OLEX2 program).[12] The cuboid can be inscribed in a sphere with a diameter of ~ 2.0 nm. Therefore, only 1 cation can fit in the small mesopores (d = 2.8 nm, V ~ 11.5 nm^3) and only 4 cations (in a T$_d$ array) can fit in the large mesopores (d = 3.5 nm, V ~ 22.4 nm^3). These simple geometrical considerations set an upper limit of 1x16 + 4x8 = 48 cations that can be exchanged in the pores of the host MOF, *i.e.* 9% cation exchange (48/544*100 ~ 9%).

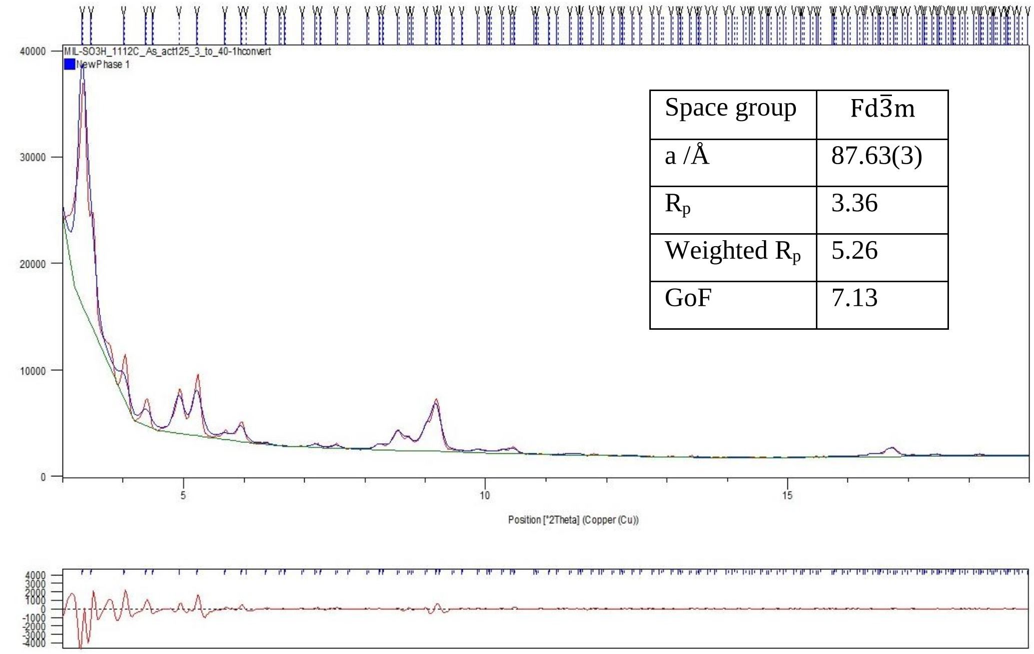

Space group	$Fd\bar{3}m$
a /Å	87.63(3)
R_p	3.36
Weighted R_p	5.26
GoF	7.13

Figure S4. Le Bail fit of PXRD pattern for desolvated **1-SO$_3$H** (activation at 125 °C for 20 h). Experimental pattern (red), calculated pattern (blue), difference pattern expt-calc (bottom). Unit cell dimensions determined from the fit and agreement indices are listed in the inset.

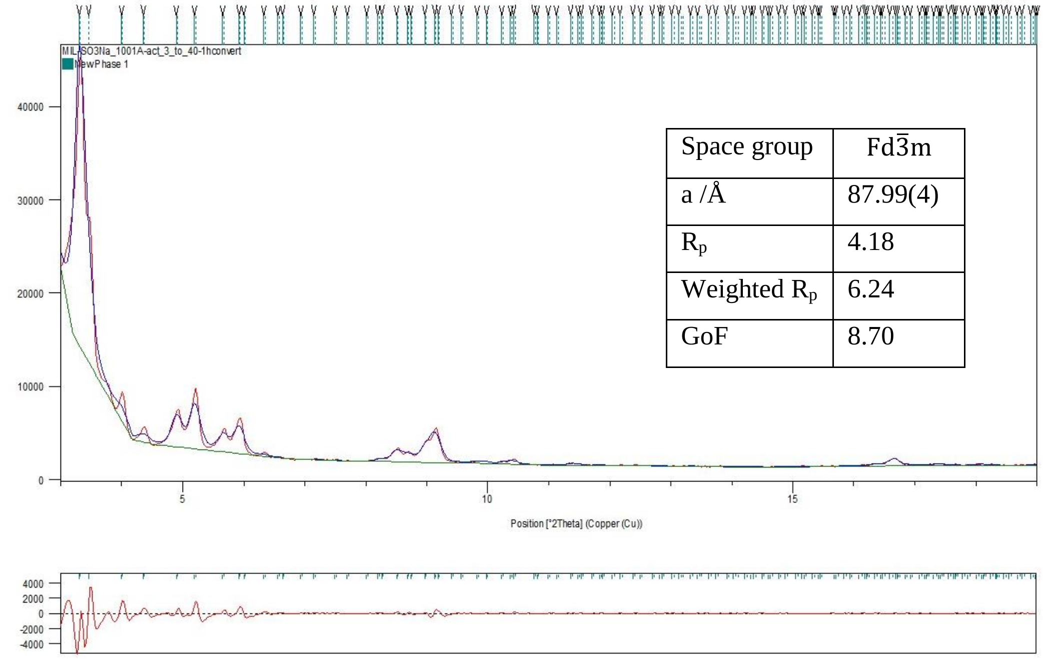

Space group	$Fd\bar{3}m$
a /Å	87.99(4)
R_p	4.18
Weighted R_p	6.24
GoF	8.70

Figure S5. Le Bail fit of PXRD pattern for desolvated **1-SO$_3$Na** (activation at 125 °C for 20 h). Experimental pattern (red), calculated pattern (blue), difference pattern expt-calc (bottom). Unit cell dimensions determined from the fit and agreement indices are listed in the inset.

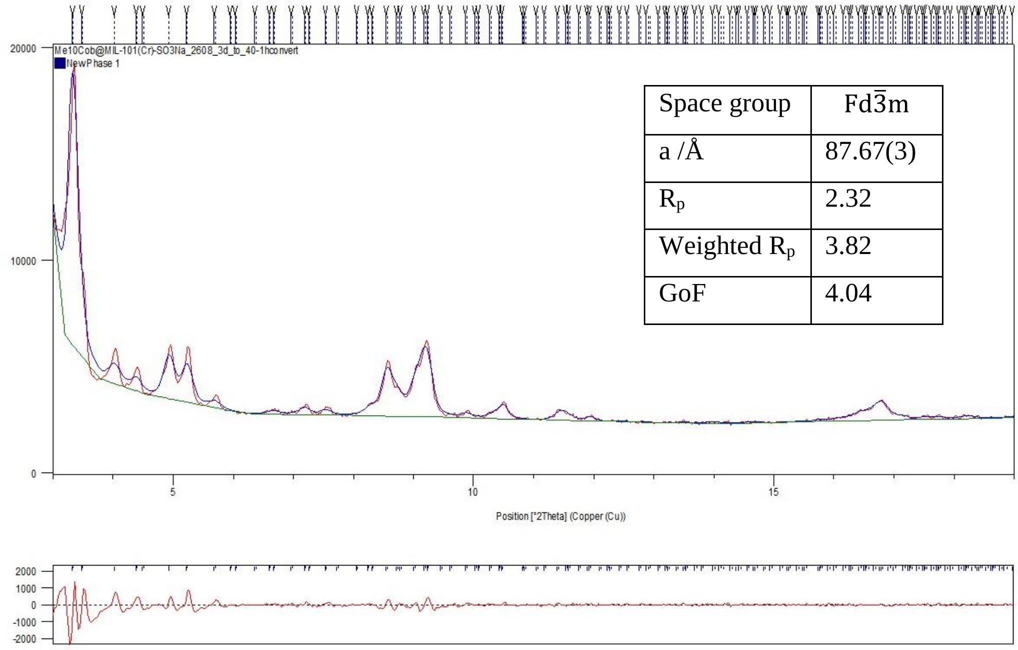

Space group	$Fd\bar{3}m$
a /Å	87.67(3)
R_p	2.32
Weighted R_p	3.82
GoF	4.04

Figure S6. Le Bail fit of PXRD pattern for **$[Cp^*_2Co]$@1-SO_3Na**. Experimental pattern (red), calculated pattern (blue), difference pattern expt-calc (bottom). Unit cell dimensions determined from the fit and agreement indices are listed in the inset.

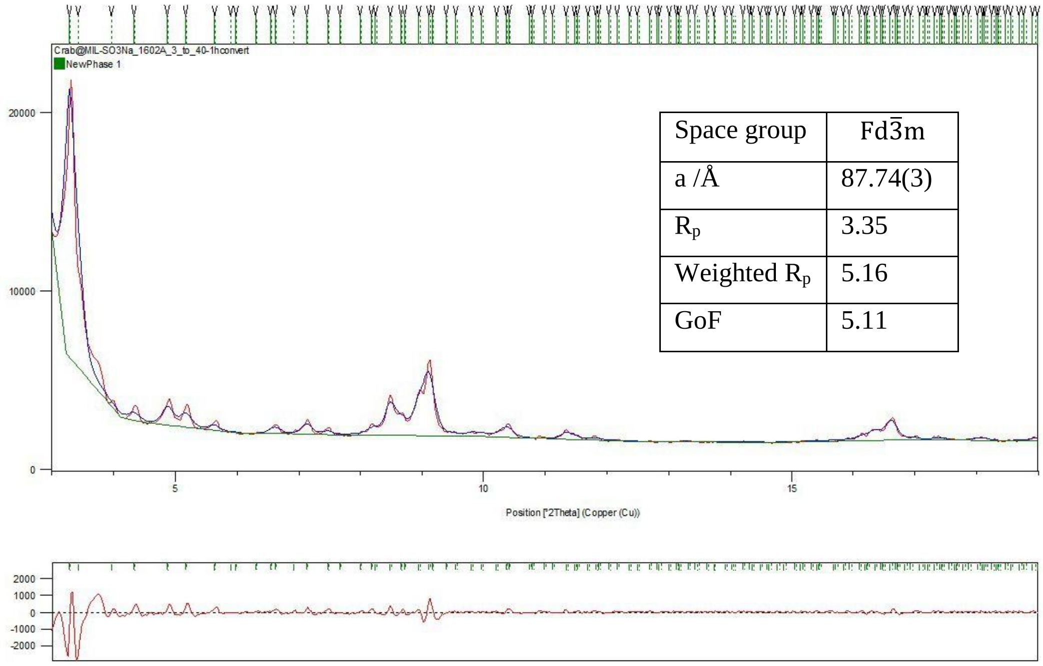

Space group	$Fd\bar{3}m$
a /Å	87.74(3)
R_p	3.35
Weighted R_p	5.16
GoF	5.11

Figure S7. Le Bail fit of PXRD pattern for **2@1-SO_3Na**. Experimental pattern (red), calculated pattern (blue), difference pattern expt-calc (bottom). Unit cell dimensions determined from the fit and agreement indices are listed in the inset.

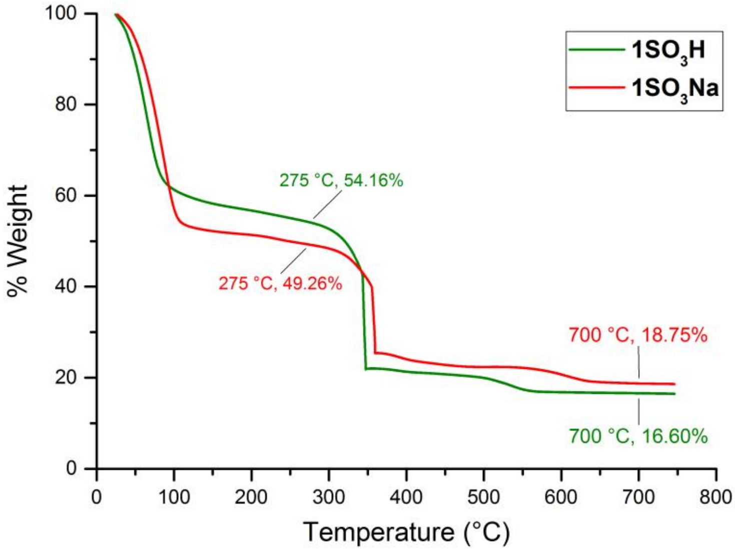

Figure S8. TGA graphs of **1-SO_3H** (green) and **1-SO_3Na** (red). The latter shows a higher inorganic/organic ratio due to encapsulation of Na^+ cations. Weight loss at 275 °C corresponds to water trapped within the pores in the as synthesized MOFs and lies in good agreement with elemental analysis (Table S1).

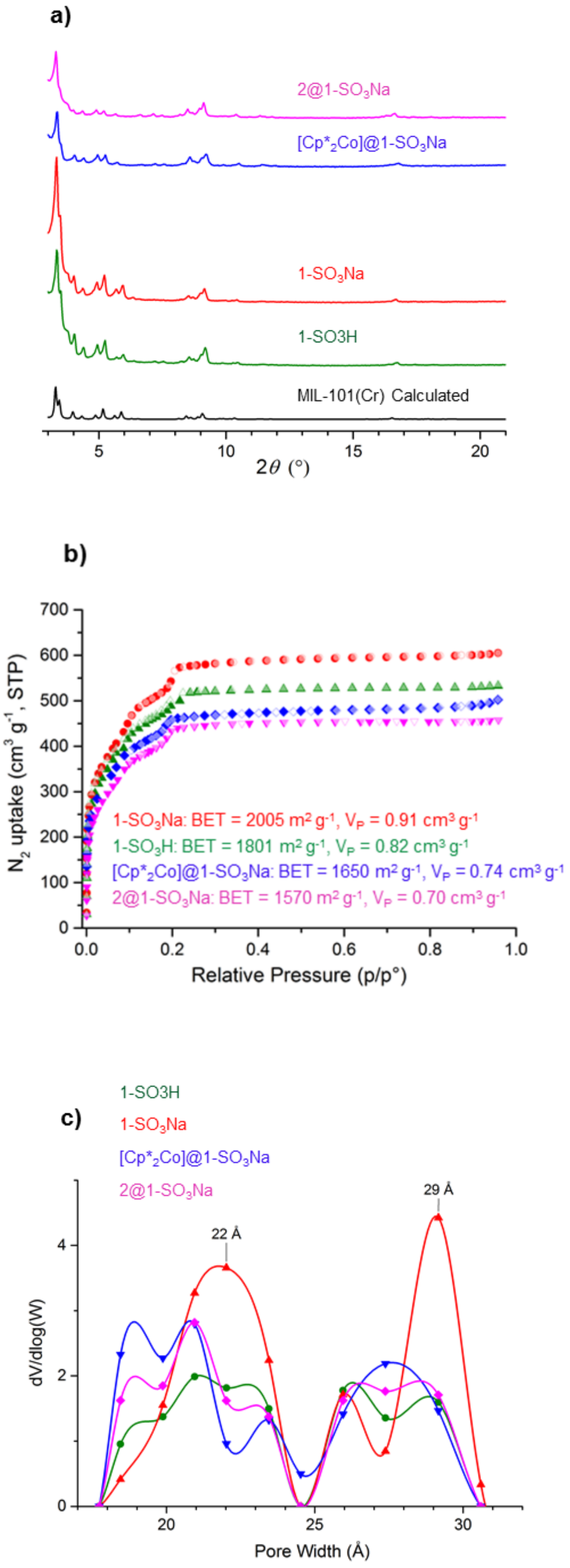

Figure S9. a) Comparison of PXRD patterns for all materials b) N_2 adsorption-desorption isotherms at 77 K for all materials after activation at 125 °C. c) Pore size distribution for all materials (Tarajona's NLDFT model for cylindrical pores).[3]

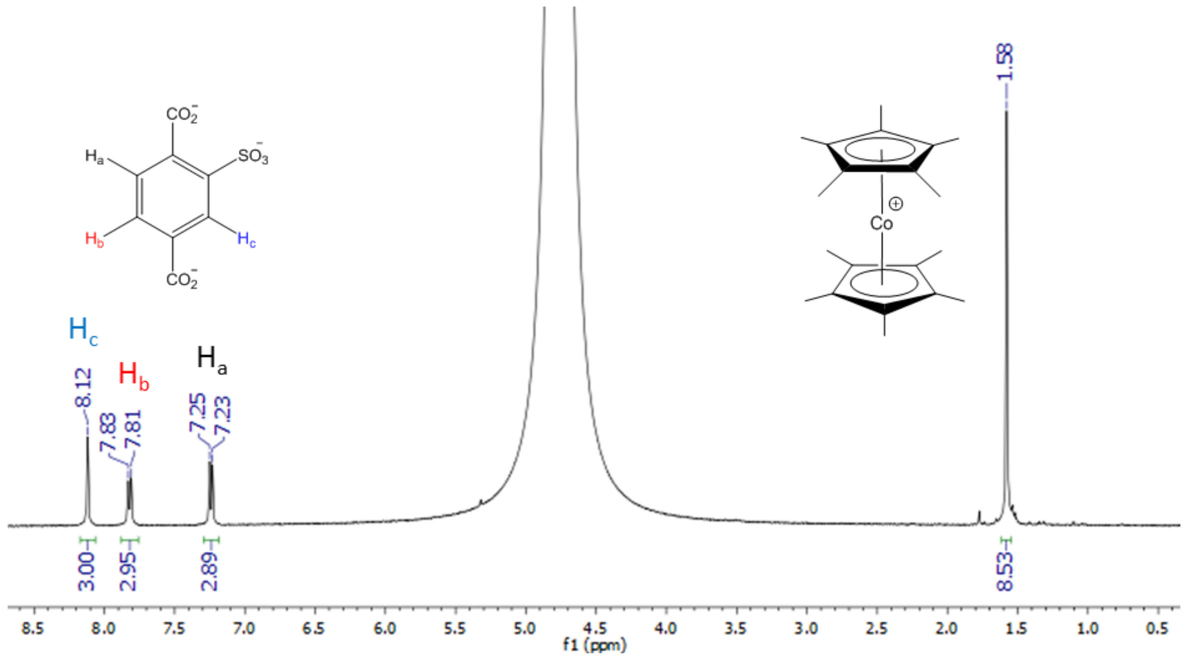

Figure S10. ^{1}H NMR spectrum of $[(Cp^{*}_{2}Co)_{0.3}Na_{1.7}][Cr_3(\mu_3\text{-}O)(BDC\text{-}SO_3)_3].46(H_2O)$ (**$[Cp^{*}_{2}Co]^{+}$@1-SO$_3$Na**) after digestion in 0.5 M NaOD (400 MHz, 298 K). Low field peaks at δ_H = 8.12, 7.82, 7.24 originate from the BDC-SO_3 linker (9H, aromatic) whereas the high field peak at δ_H = 1.58 arises from the encapsulated $[Cp^{*}_{2}Co]^{+}$ cation (8.5H, Me groups). Integration of the respective peaks shows that approximately 14 % of the charge balancing cations have been exchanged (x = 8.5/30 = 0.28 out of a total of 2 cations per formula unit) in very good agreement with analytical data (Table S2).

1-SO_3H

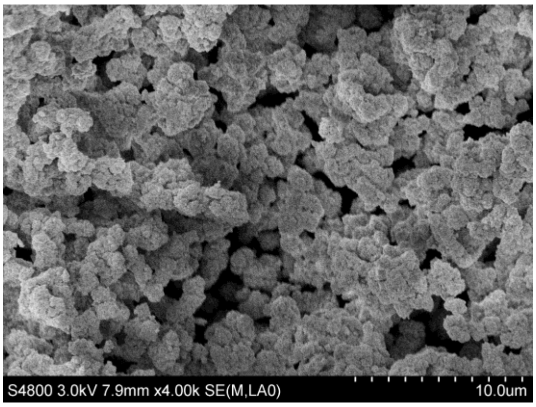

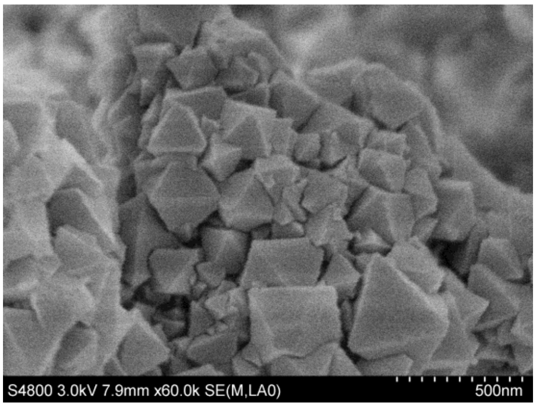

1-SO_3Na

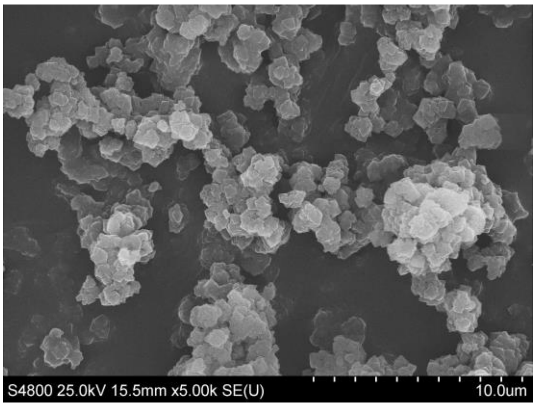

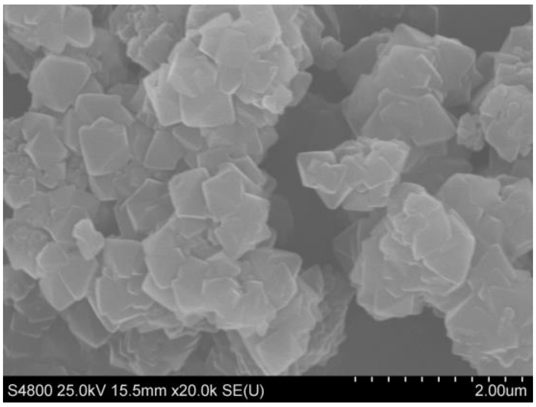

Figure S11. SEM images of as synthesized **1-SO_3H** (top), **1-SO_3Na** (bottom). Particles retain their morphology and size distribution (0.2 – 1 μm).

$[Cp^*{}_2Co]^+$@1-SO_3Na

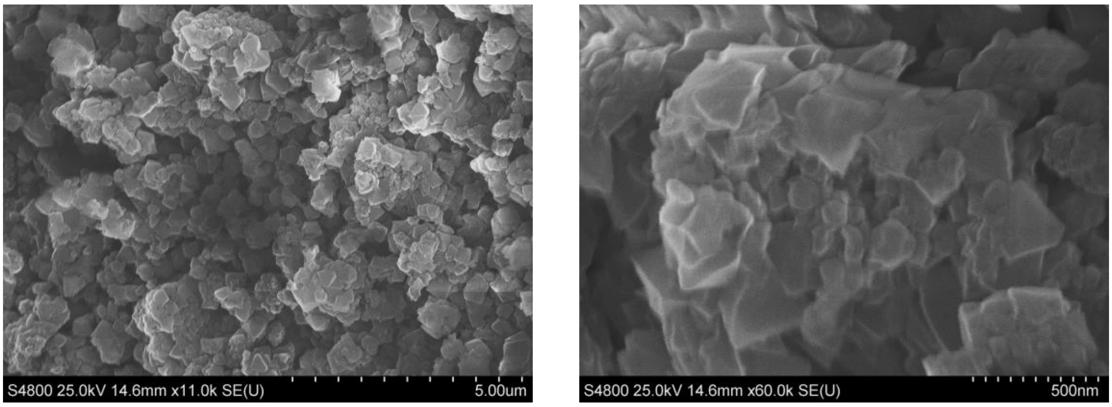

2@1-SO_3Na

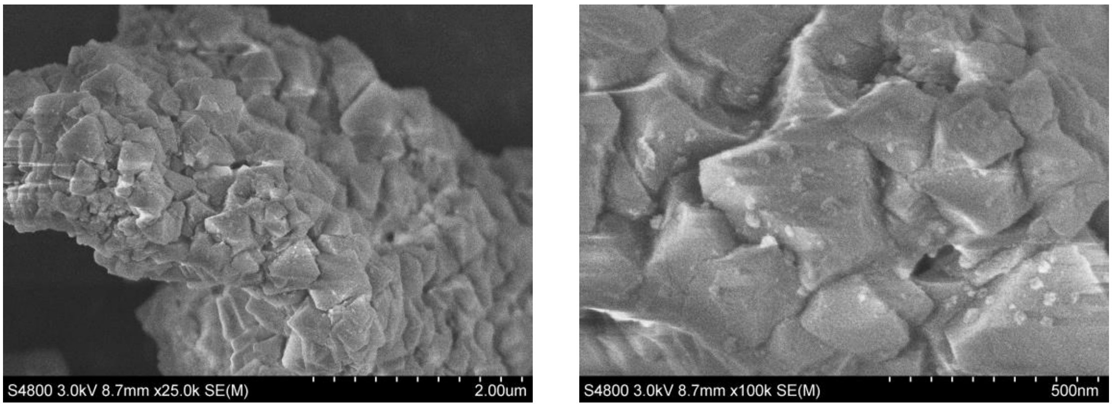

Figure S12. SEM images of as synthesized of **$[Cp^*{}_2Co]^+$@1-SO_3Na** (top) and **2@1-SO_3Na** (bottom). Particles retain their morphology and size distribution (0.2 – 1 μm).

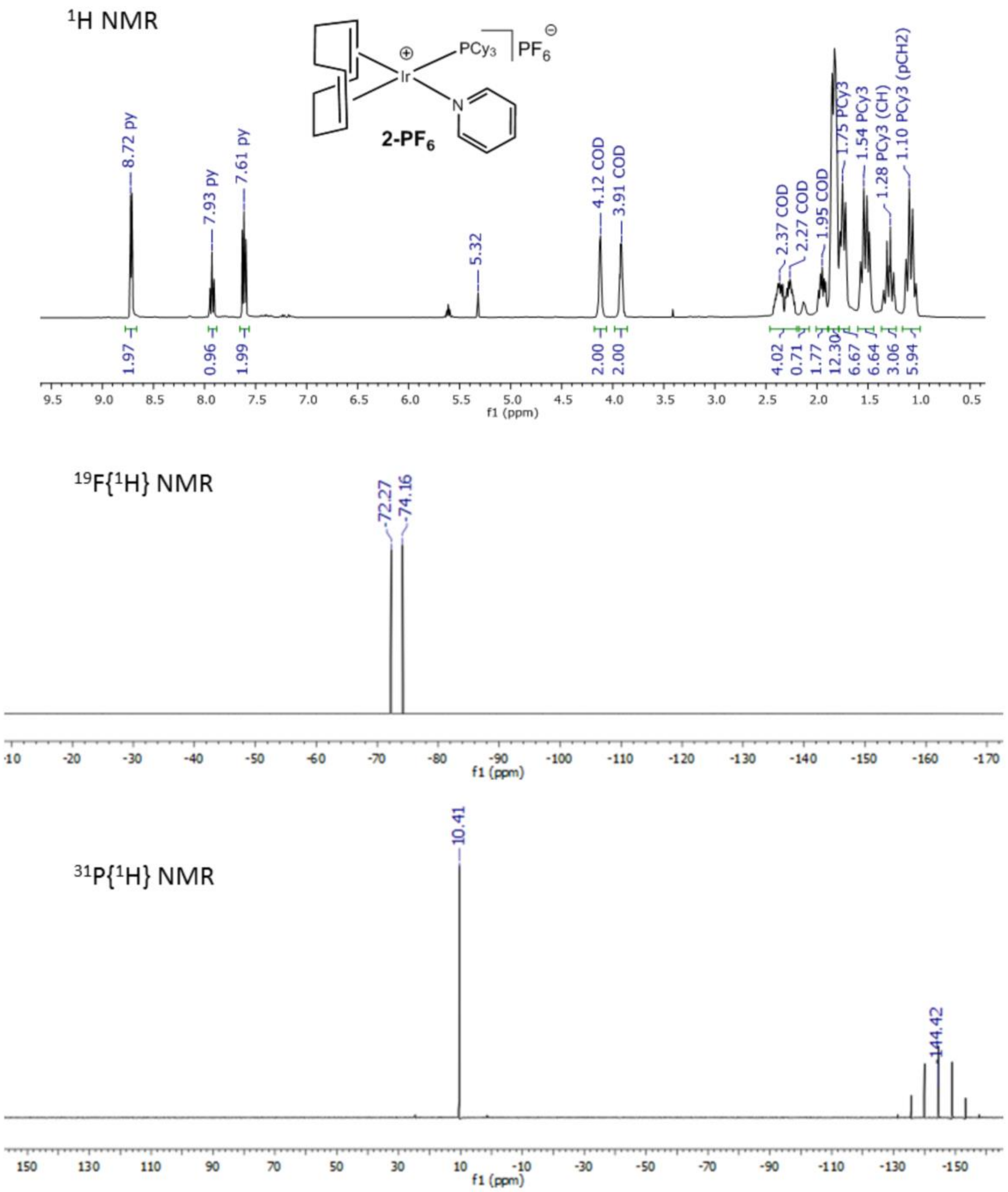

Figure S13. ^{1}H, ^{19}F{^{1}H} and ^{31}P{^{1}H} NMR spectra in CD_2Cl_2 at 298 K of **2-PF$_6$** (Crabtree's catalyst). No signals are observed in the respective ^{19}F{^{1}H} spectrum of **2@1-SO$_3$Na** after digestion in NaOD 0.5 M.

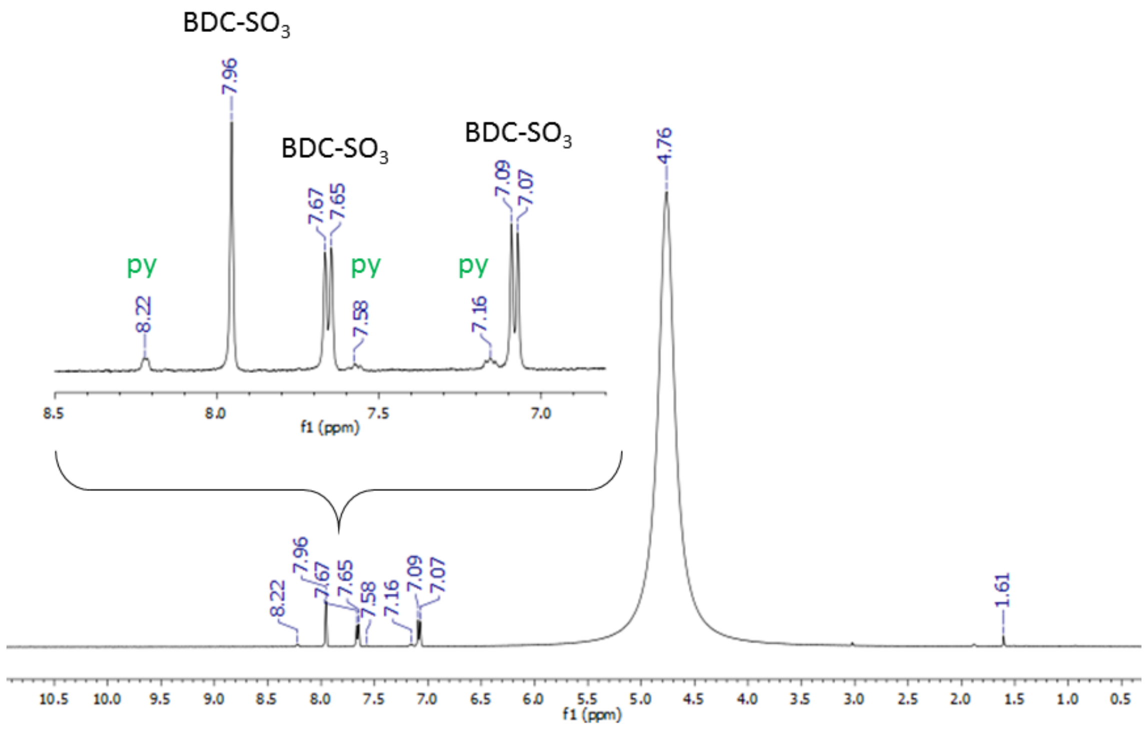

Figure S14. ^{1}H NMR of **1@SO$_3$Na** in solution after digestion in 0.5 M NaOD. Inset shows the three low intensity peaks at 8.22, 7.58 and 7.16 ppm assigned to pyridine along with peaks originating from the BDC-SO$_3$ linker.

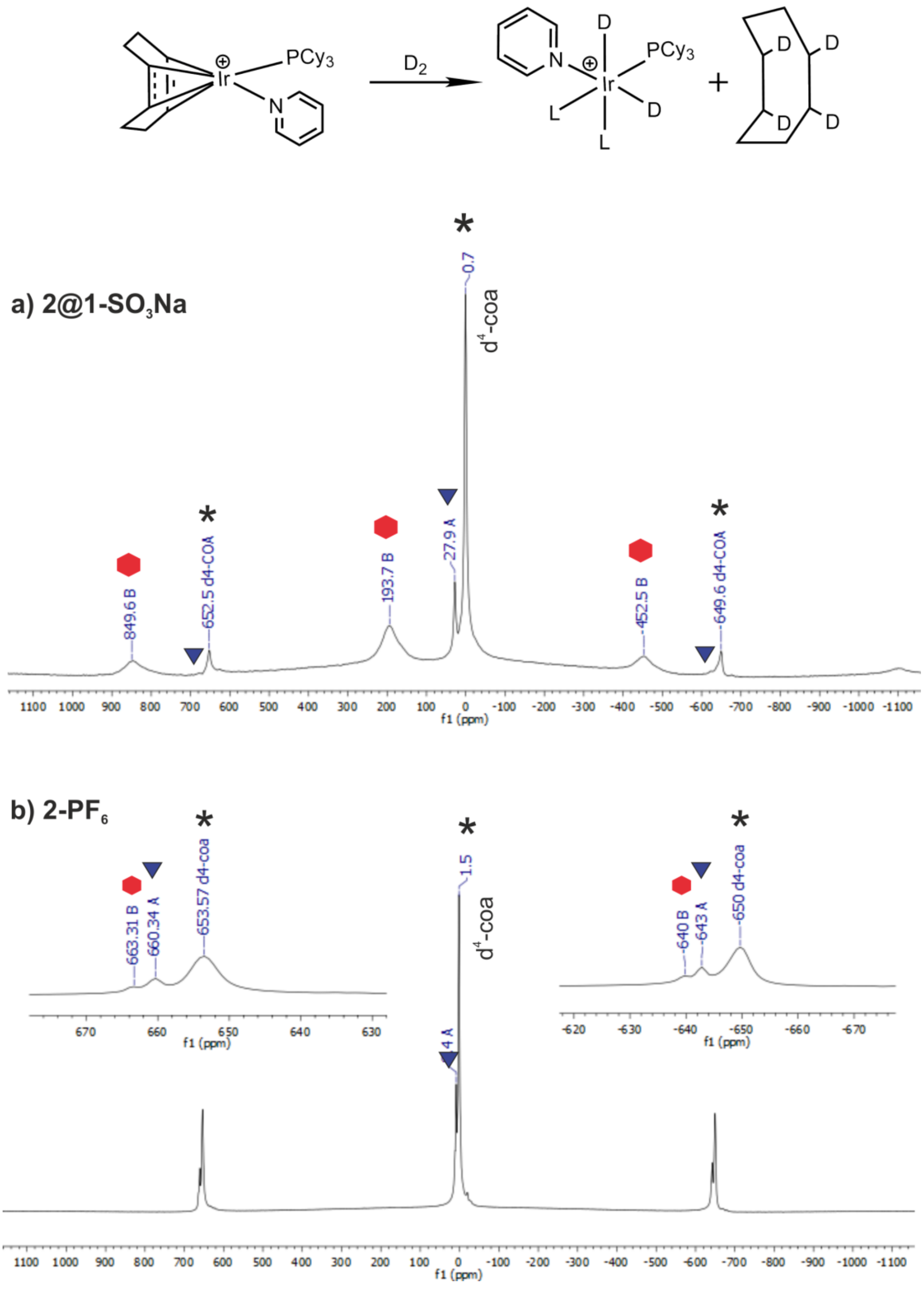

Figure S15. ^{2}H solid-state NMR at a MAS rate of 40 KHz after deuteration of a) **2@1-SO$_3$Na** and b) **2-PF$_6$** in a gas/solid reaction. Main peak corresponds to d$_4$-cycloctane (d$_4$-coa, asterisk) being formed due to deuteration of the cod ligand. For both systems, two more currently unknown species are observed, labelled as A (blue triangle) and B (red hexagon).

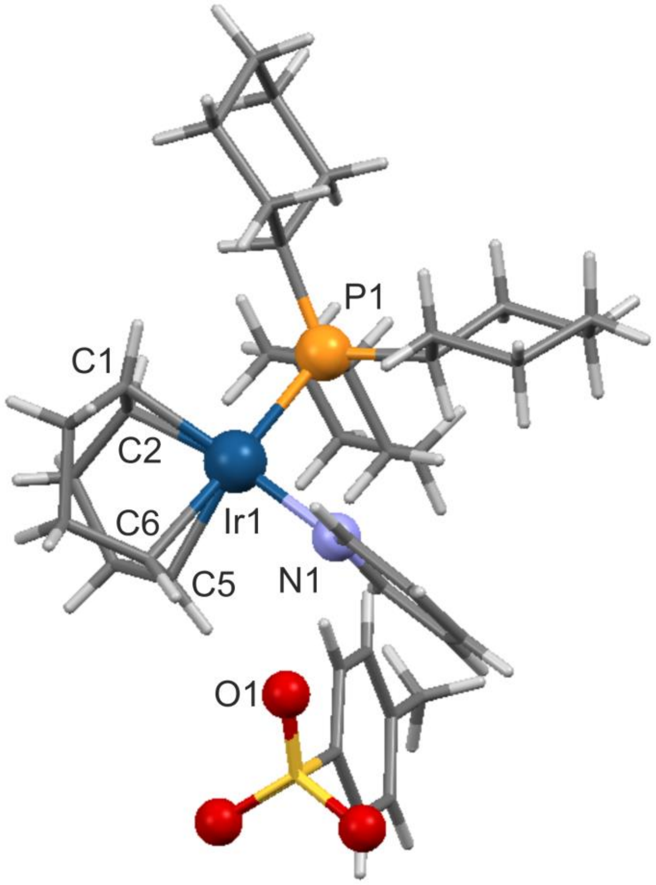

Figure S16. Single crystal structure of **2-OTs** in which pyridine is coordinated to Ir (*cis* to PCy_3) and tosylate (OTs) acts as the counter anion. Selected bond lengths (Å) and angles (°): Ir-P1 2.3378(6), Ir1-N1 2.105(2), Ir1-C1 2.135(3), Ir1-C2 2.148(3), Ir1-C5 2.183(2), Ir1-C6 2.235(2), C1-C2 1.405(4), C5-C6 1.390(4), Ir1-O1 4.478, N1-Ir1-P1 94.15(6). Refer to Tables S4 and S5 for more details.

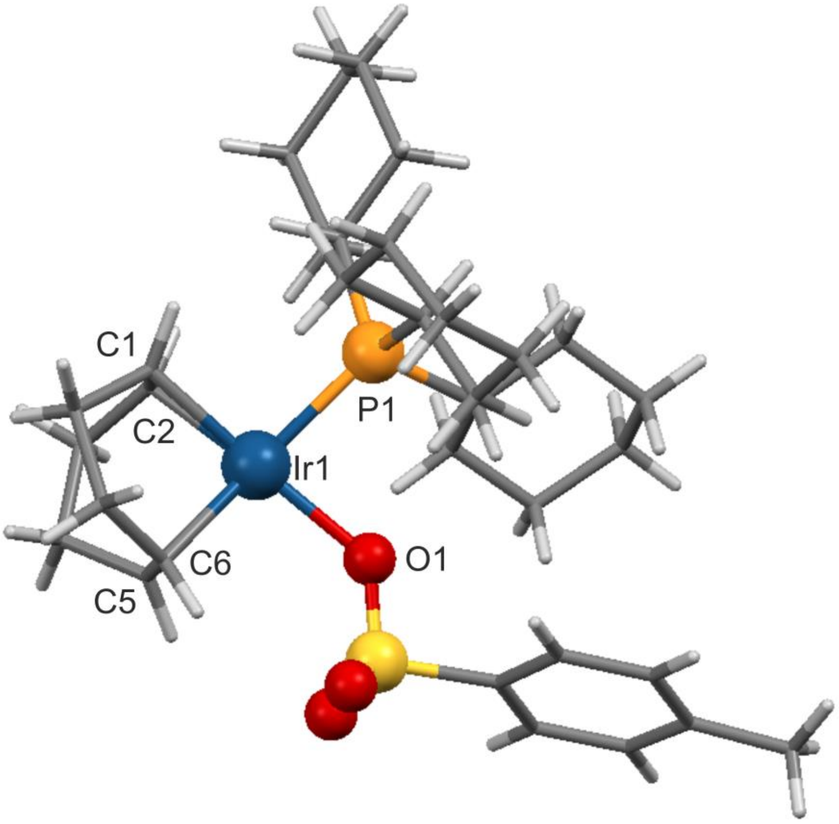

Figure S17. Single crystal structure of **3** in which tosylate (OTs) is coordinated to Ir, occupying the coordination site *cis* to PCy_3. Selected bond lengths (Å) and angles (°): Ir-P1 2.3391(9), Ir1-O1 2.105(2), Ir1-C1 2.105(3), Ir1-C2 2.096(3), Ir1-C5 2.226(3), Ir1-C6 2.198(3), C1-C2 1.429(6), C5-C6 1.390(6), O1-Ir1-P1 83.95(7). Refer to Tables S4 and S5 for more details.

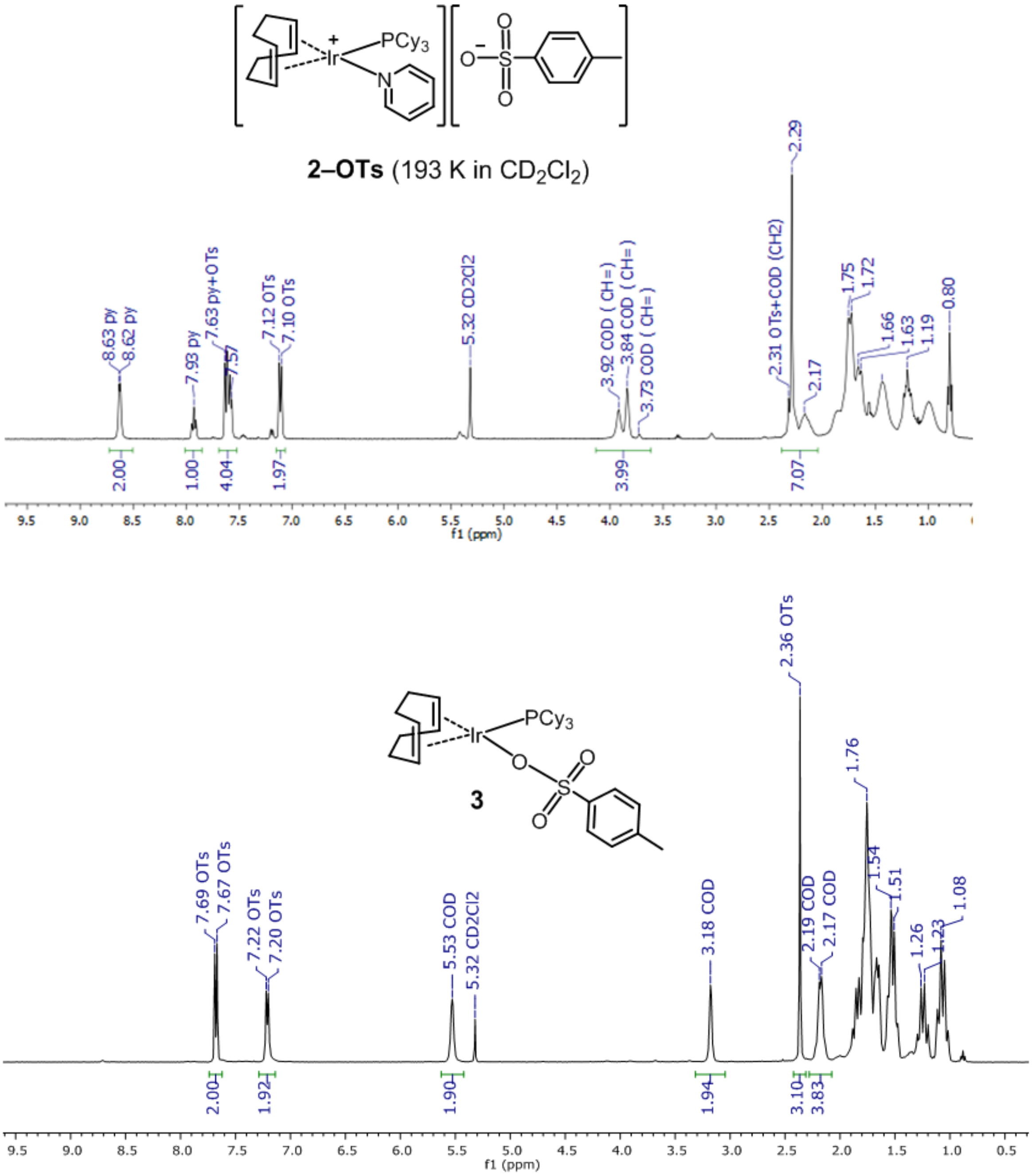

Figure S18. ^{1}H NMR spectra of **2-OTs** at 193 K (top) and **3** at 298 K (bottom) in CD_2Cl_2. Olefinic protons of the cod ligand in each complex show characteristically different chemical shifts. Complex **2-OTs** (selected resonances): δ_H = 8.63 (ortho-H Py), 7.93 (para-H Py), 7.63-7.57 (OTs and meta-H Py), 7.11 (d, J_{HH} = 7.9 Hz, OTs), 3.92 – 3.73 (4H, CH cod), 2.33-2.09 (overlapping CH_2 cod and CH_3 OTs). Complex **3** (selected resonances): δ_H = 7.68 (d, J_{HH} = 8 Hz, OTs), 7.21 (d, J_{HH} = 7.6 Hz, OTs), 5.53 (2H, CH cod), 3.18 (2H, CH cod), 2.36 (CH_3 OTs).

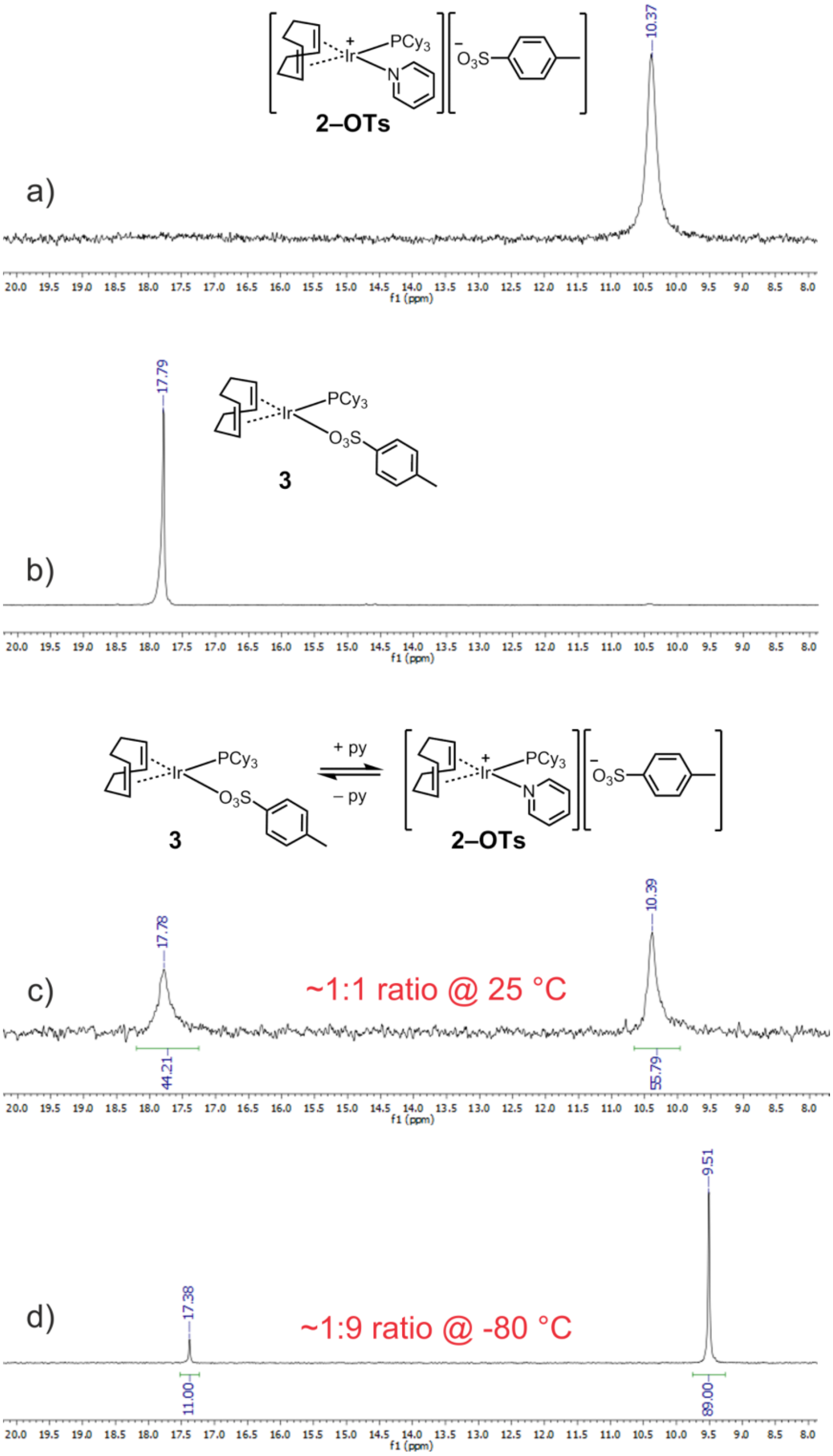

Figure S19. $^{31}P\{^{1}H\}$ NMR spectra in CD_2Cl_2 of: a) Complex **2-OTs** in one equivalent excess of pyridine; b) Complex **3**; c) Complex **2-OTs** after removing excess pyridine at 298 K and d) after cooling down at 193 K. Removal of excess pyridine leads to formation of **3**, showing a dynamic and reversible ligand exchange process between **2-OTs** and **3** (1:1 ratio at 298 K which shifts to 9:1 in favor of **2-OTs** at 193 K). Addition of pyridine in a CD_2Cl_2 solution of **3** restores **2-OTs**.

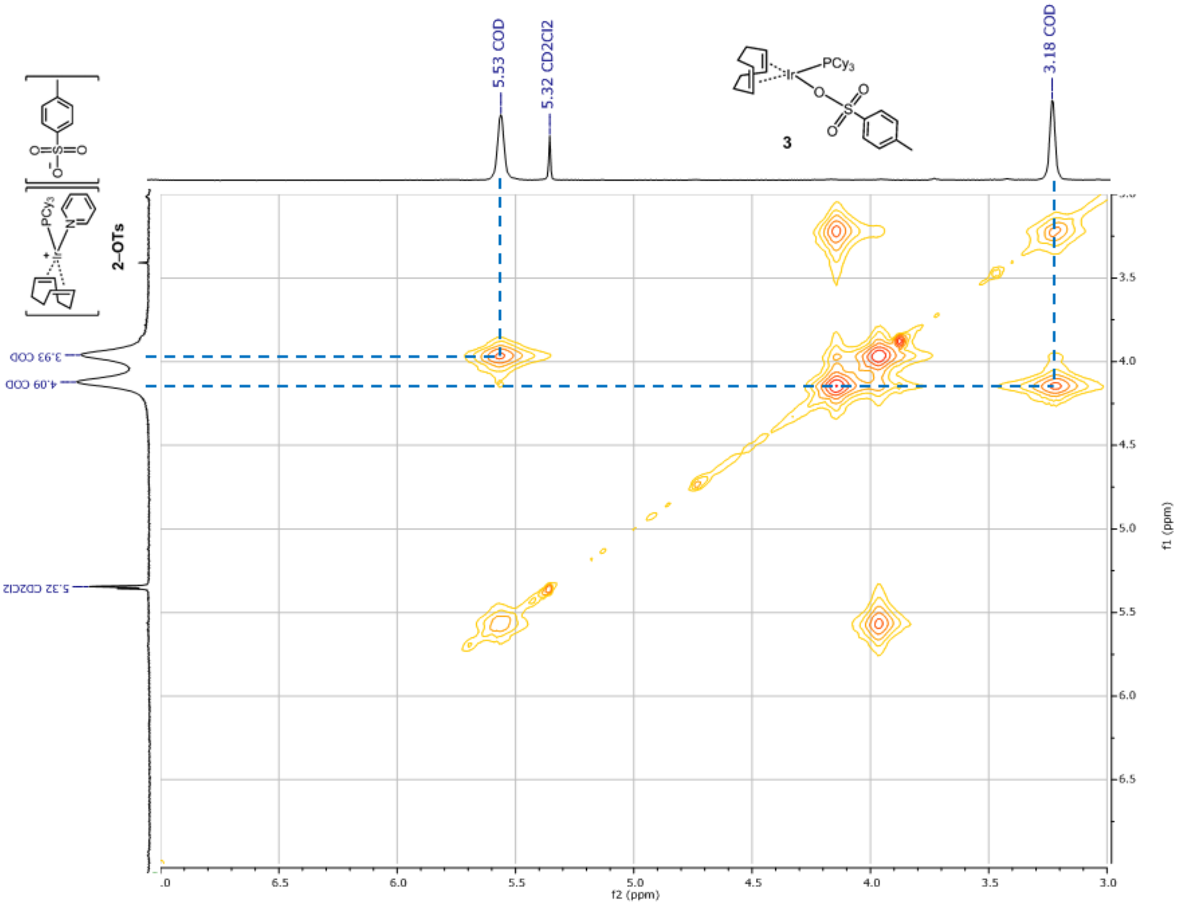

Figure S20. ^{1}H NMR EXSY (CD_2Cl_2, 298 K) spectrum showing cross-peaks between the olefinic proton signals of the cod ligands in **2-OTs** (δ = 4.09, 3.93) and **3** (δ = 5.53, 3.18 ppm), indicative of a reversible equilibrium in solution between the two species.

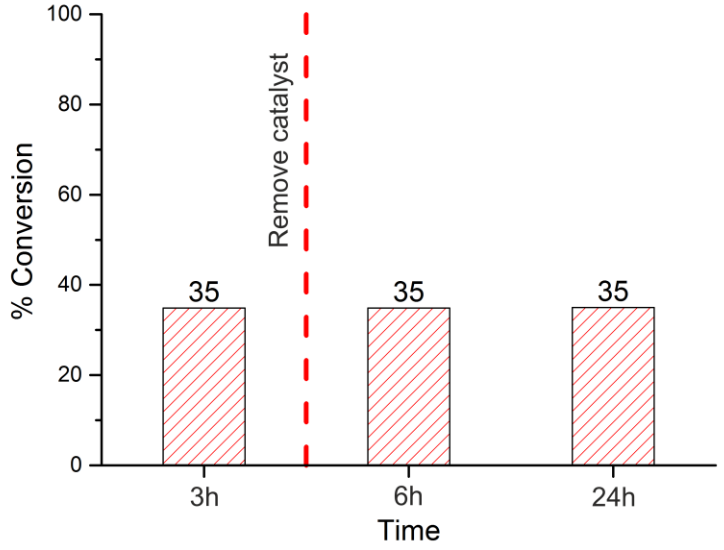

Figure S21. Leaching test: Supernatant was anaerobically separated from the heterogeneous catalyst after 3 h (red dashed line) and left to react after addition of a fresh batch of H_2 gas. Conversion did not increase any more, verifying the heterogeneity of the system. Conditions: 3.5 mgs of **2@1-SO$_3$Na**, [1-octene] = 1.0 M, V = 4 mL (100 ppm loading). Experimental details are provided on page S11.

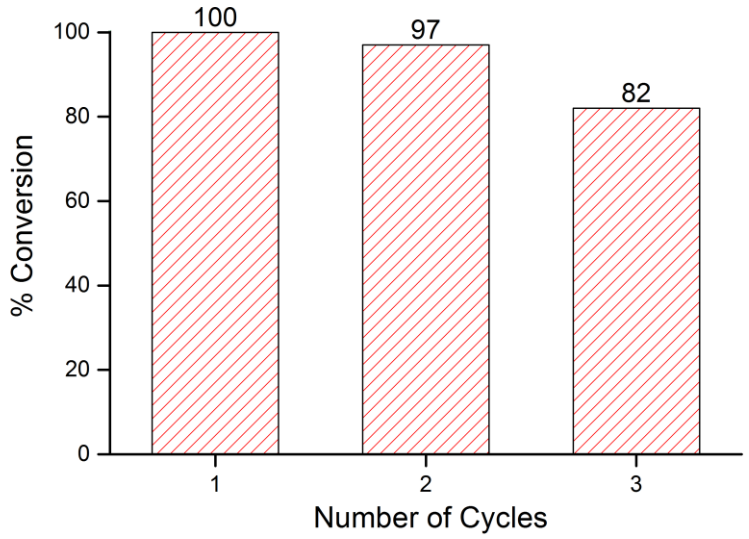

Figure S22. Recycling of **2@1-SO$_3$Na** with 1-octene as the substrate: After the end of each cycle (20 h), the supernatant was anaerobically separated from the heterogeneous catalyst by cannula-filtration and a fresh batch of 1-octene solution in CH_2Cl_2 and H_2 gas was added. Conditions: 3.5 mgs of **2@1-SO$_3$Na**, [1-octene] = 1.0 M, V = 4 mL (100 ppm loading), t = 20 h. Experimental details are provided on page S12.

a)

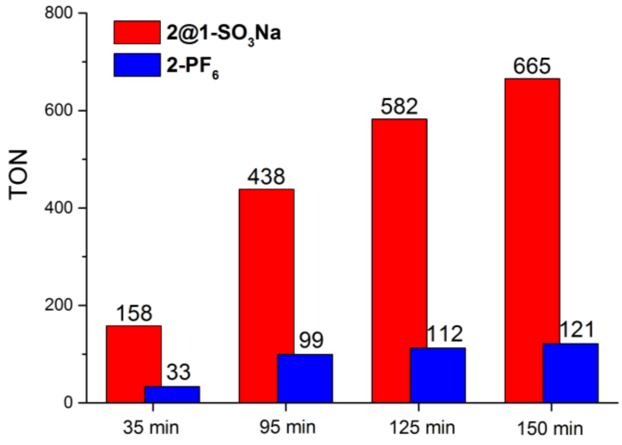

b)

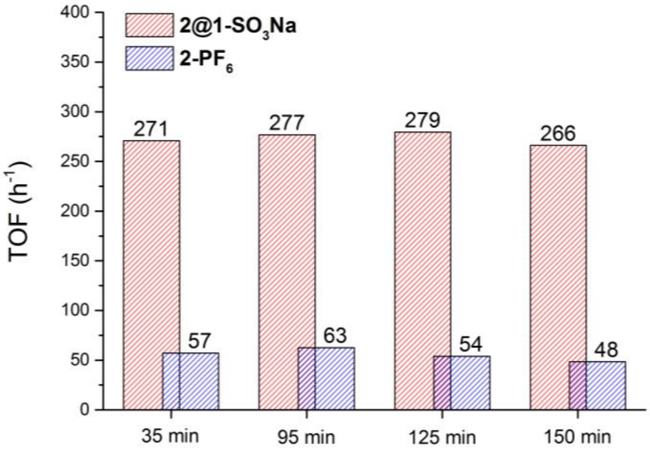

c)

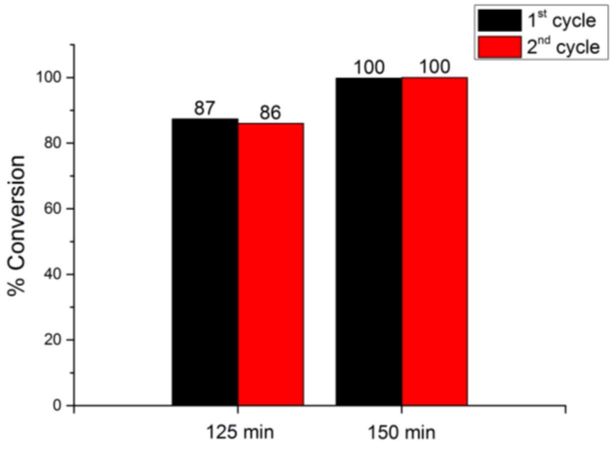

Figure S23. Gas/solid hydrogenation of 1-butene: a) Comparison of TON and b) TOF (h^{-1}) using **2@1-SO_3Na** (red) or **2-PF_6** (blue) as solid-state catalysts; c) Recycling of 1-butene with **2@1-SO_3Na**. At least one more cycle is feasible. See page S14 for experimental details.

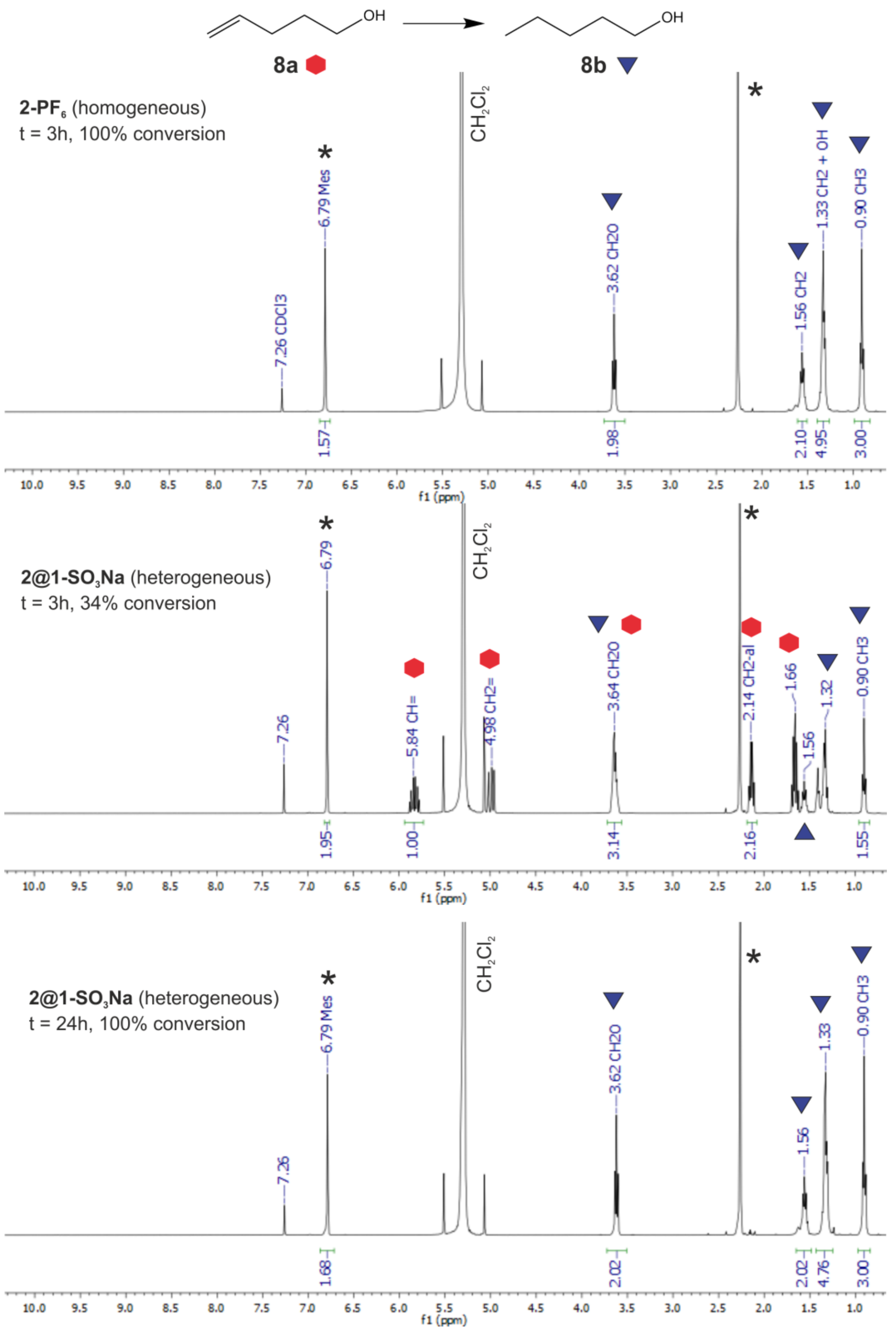

Figure S24. ^{1}H NMR spectra for hydrogenation of pent-4-en-1-ol (**8a**, red hexagons) with **2-PF$_6$** in t = 3 h (top) and **2@1-SO$_3$Na** in t = 3 h (middle) and t = 24 h (bottom). Isomerization products are not detected and 100% selectivity to hydrogenation and formation of *n*-pentanol (**8b**, blue triangles) is observed. Mesitylene (*) is used as a standard to measure mass balance (>95%).

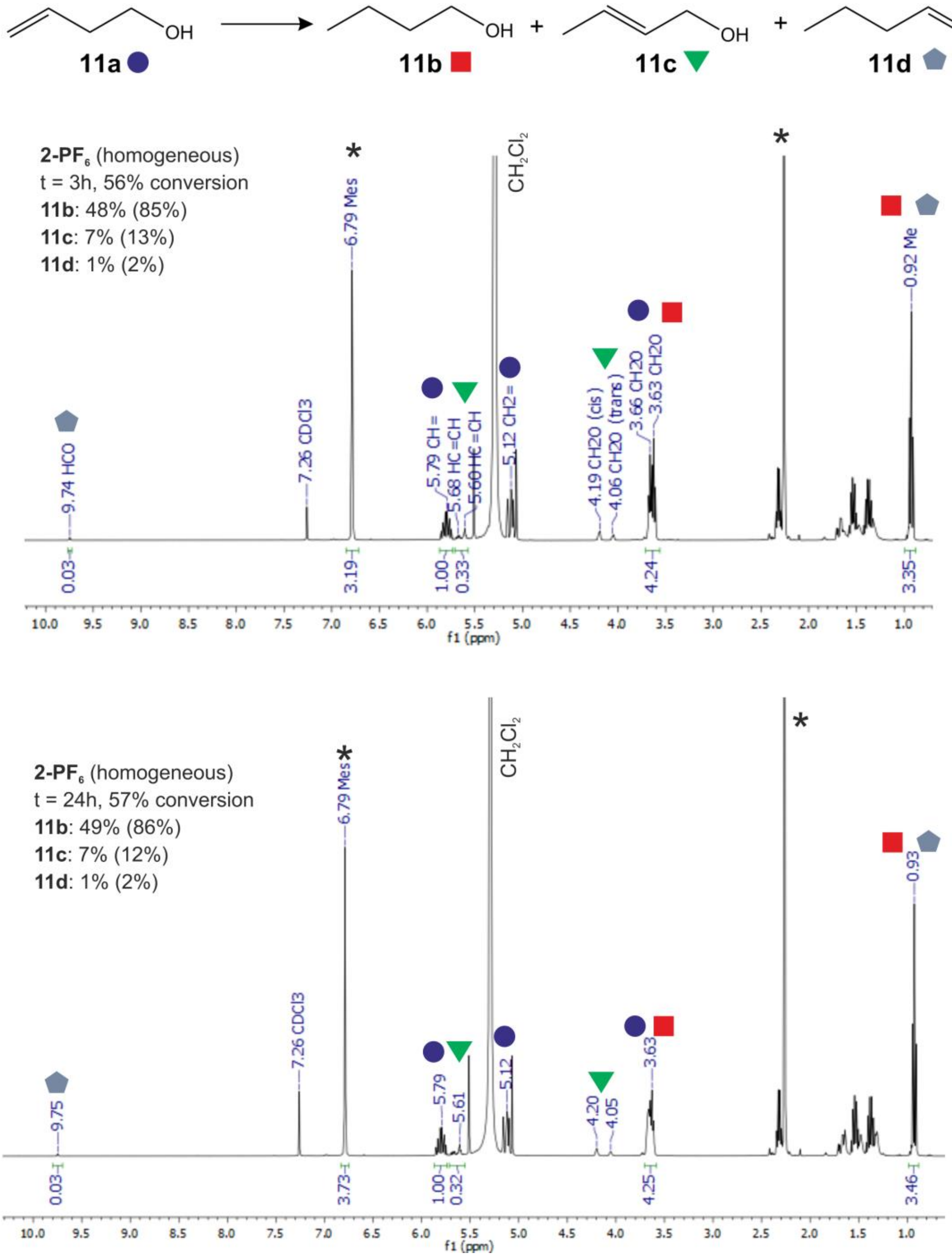

Figure S25. ^{1}H NMR spectra for homogeneous hydrogenation of but-3-en-1-ol (**11a**, blue circles) with **2-PF$_6$** in t = 3 h (top) and t = 24 h (bottom). Hydrogenation to *n*-butanol (**11b**, red squares) practically stops after 3 h. Isomerization products are also detected: crotyl alcohol (**11c**, green triangles) and butanal (**11d**, grey pentagons). Yield and selectivity (in parenthesis) for each product are shown in the inset. Mesitylene (*) is used as a standard to measure mass-balance (>95%).

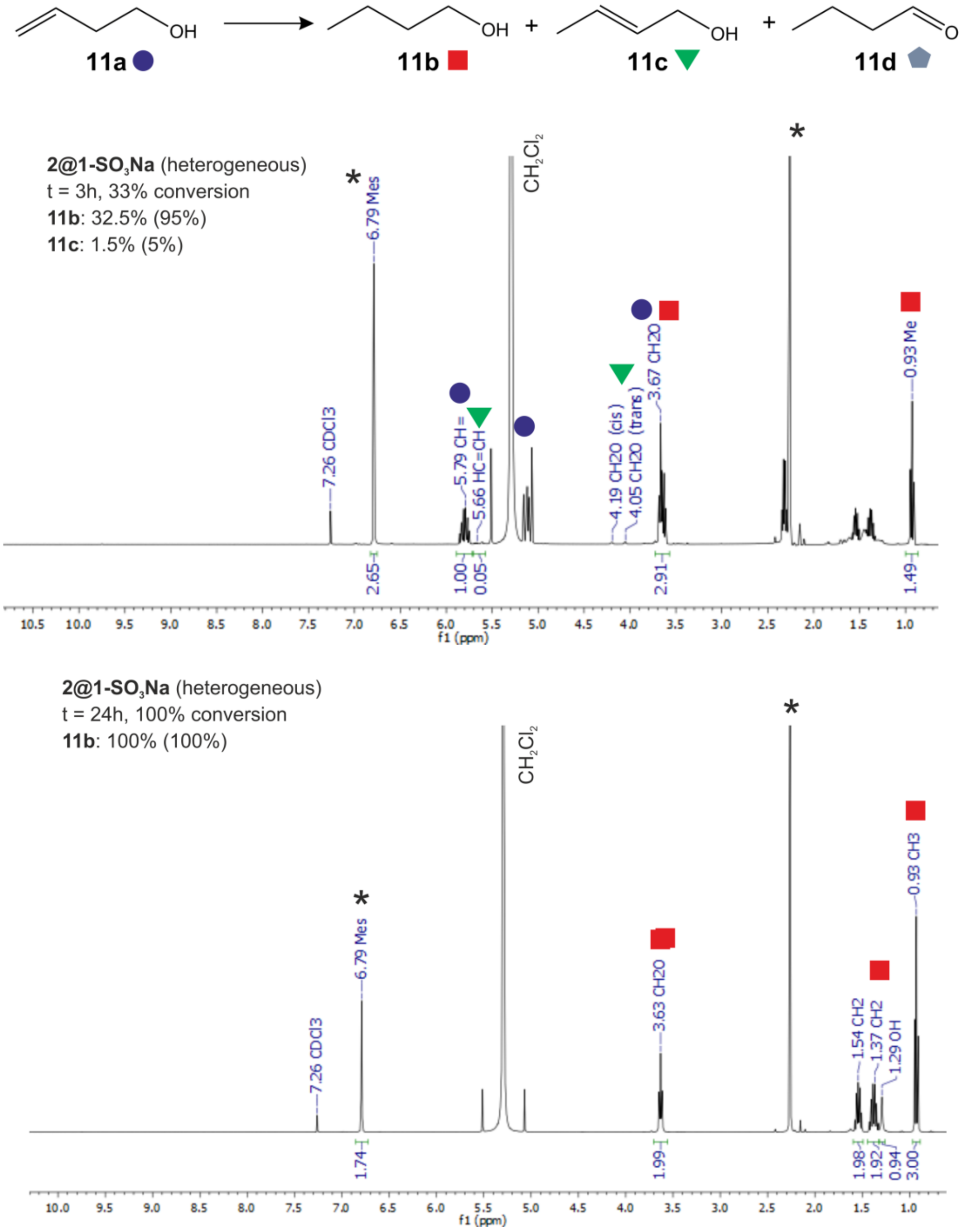

Figure S26. ^{1}H NMR spectra for heterogeneous hydrogenation of but-3-en-1-ol (**11a**, blue circles) with **2@1-SO$_3$Na** in t = 3 h (top) and t = 24 h (bottom). Traces of **11c** (green triangles) are only detected after 3 h and 100% conversion is observed after 24 h exclusively to *n*-butanol (**11b**, red squares). Yield and selectivity (in parenthesis) for each product are shown in the inset. Mesitylene (*) is used as a standard to measure mass-balance (>95%).

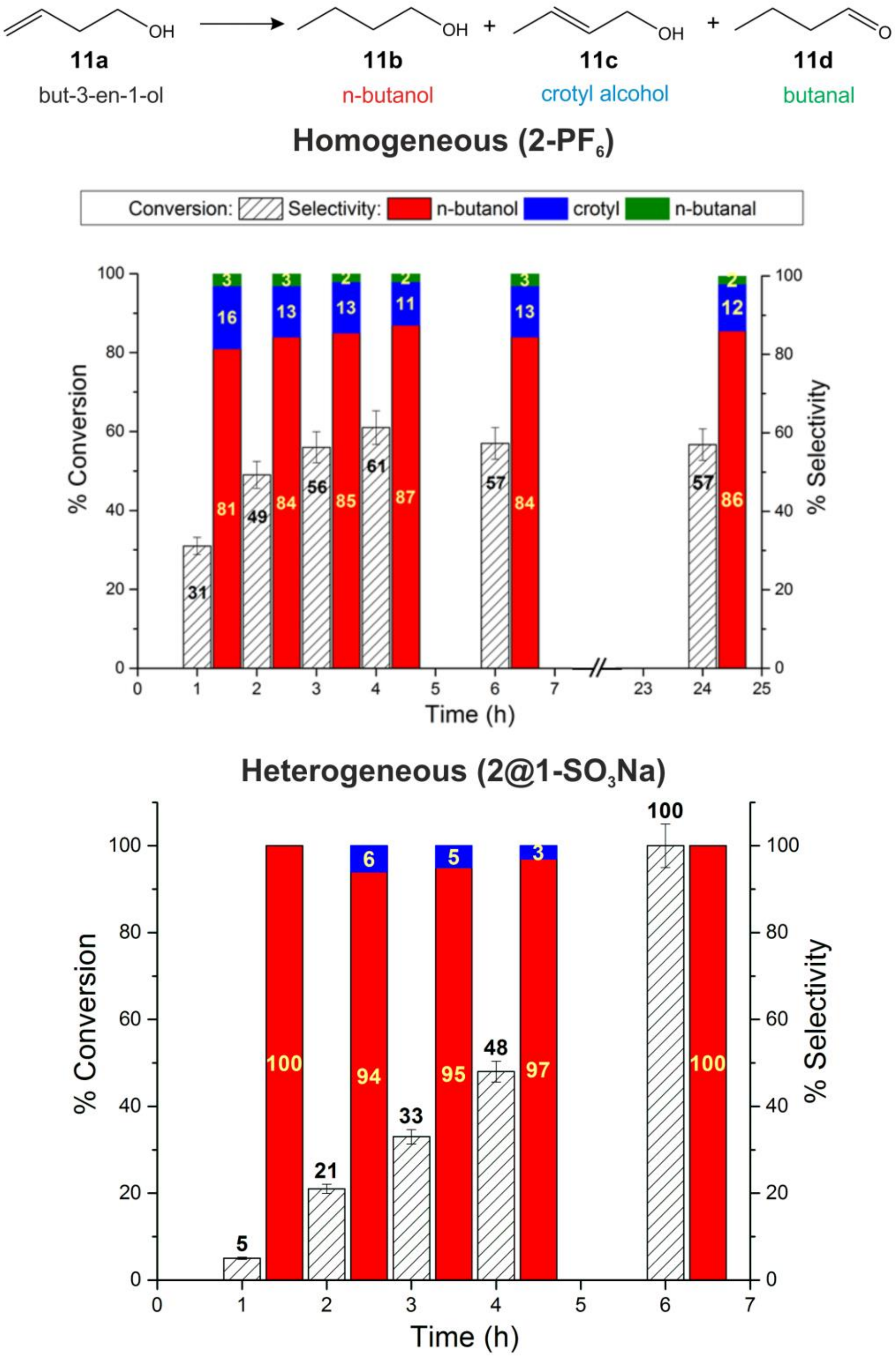

Figure S27. Conversion and selectivity (in colour) time profile for the hydrogenation of but-3-en-1-ol (**11a**) with homogeneous **2-PF6** (top) or heterogeneous **2@1-SO3Na** (bottom) catalysts. **2-PF6** is practically deactivated in 3 h. By contrast, **2@1-SO3Na** remains productive and conversion reaches 100% in 6 h with 100% selectivity to hydrogenation and formation of n-butanol.

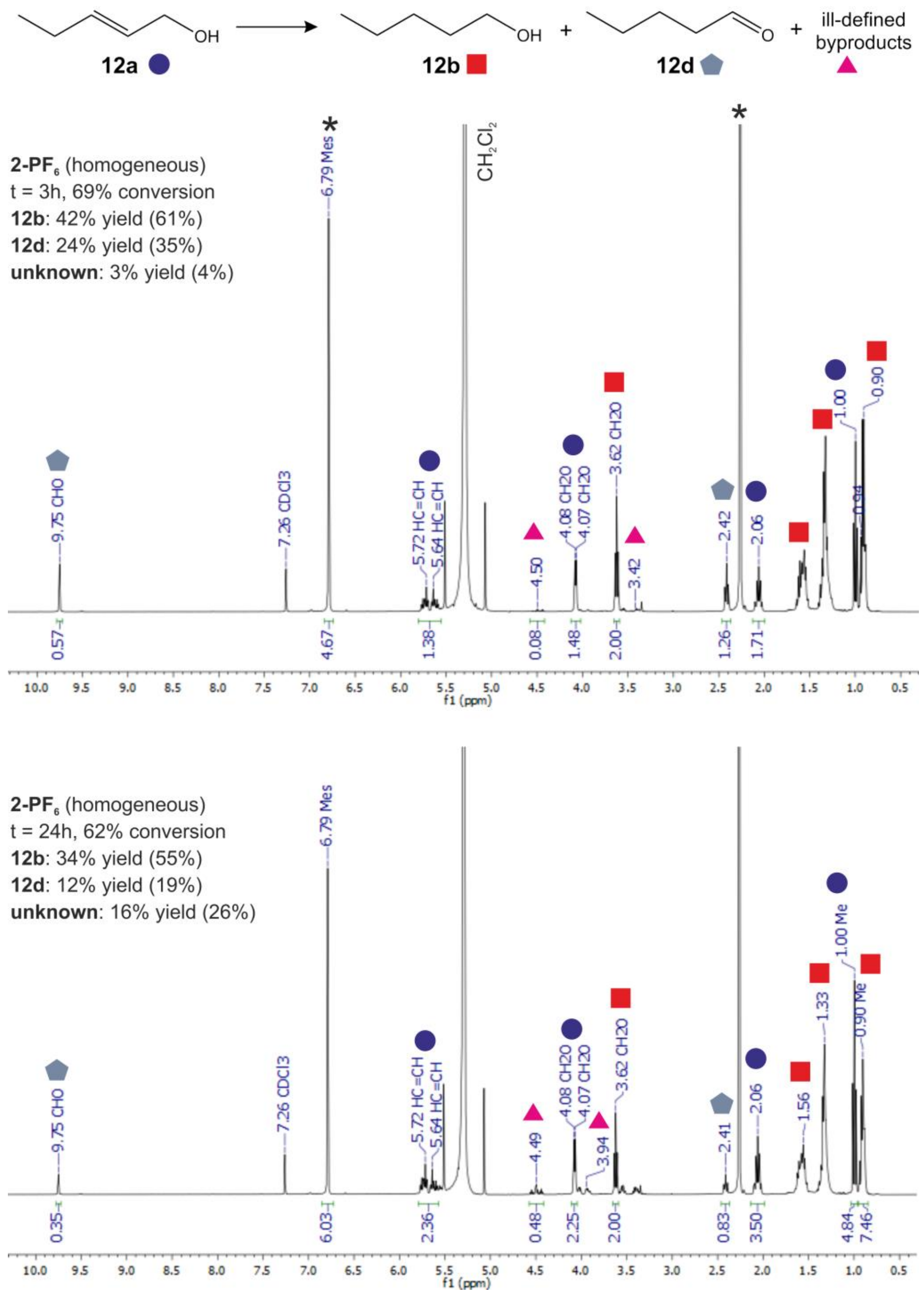

Figure S28. ^{1}H NMR spectra for homogeneous hydrogenation of *trans* pent-2-en-1-ol (**12a**, blue circles) with **2-PF$_6$** in t = 3 h (top) and t = 24 h (bottom). Turnover stops after 3 h at 69% conversion with only 61% selectivity to hydrogenation and formation of *n*-pentanol (**12b**, red squares). Ill-defined condensation products are also detected, particularly after 24 h (magenta triangles). Yield and selectivity (in parenthesis) for each product are shown in the inset. Mesitylene (*) is used as a standard to measure mass-balance (>95%).

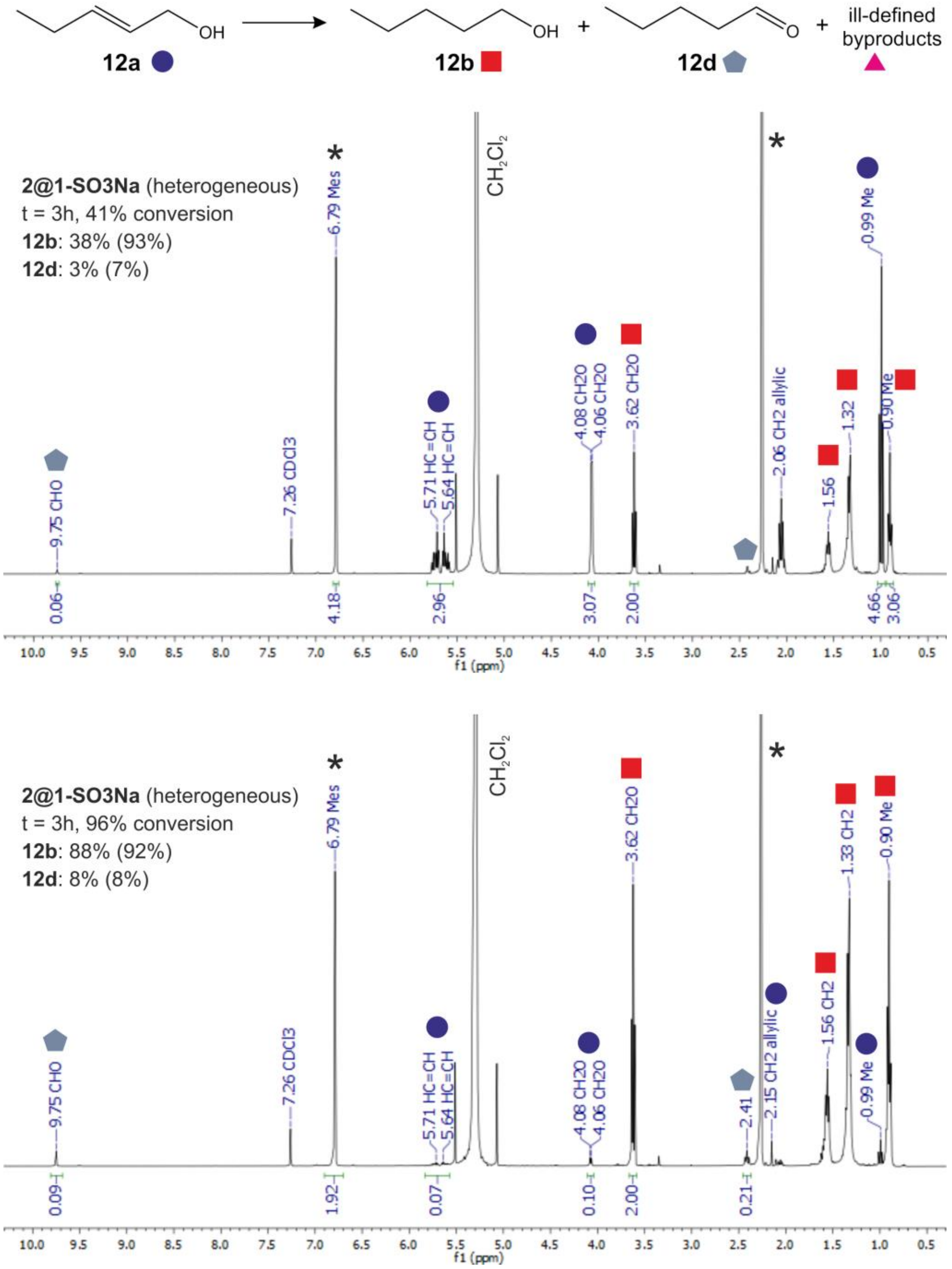

Figure S29. ^{1}H NMR spectra for heterogeneous hydrogenation of pent-2-en-1-ol (**12a**, blue circles) with **2@1-SO$_3$Na** in t = 3 h (top) and t = 24 h (bottom). Conversion reaches 96% in 24 h with high selectivity (> 90%) to hydrogenation and formation of n-pentanol (**12b**, red squares). No other products are detected. Yield and selectivity (in parenthesis) for each product are shown in the inset. Mesitylene (*) is used as a standard to measure mass-balance (>95%).

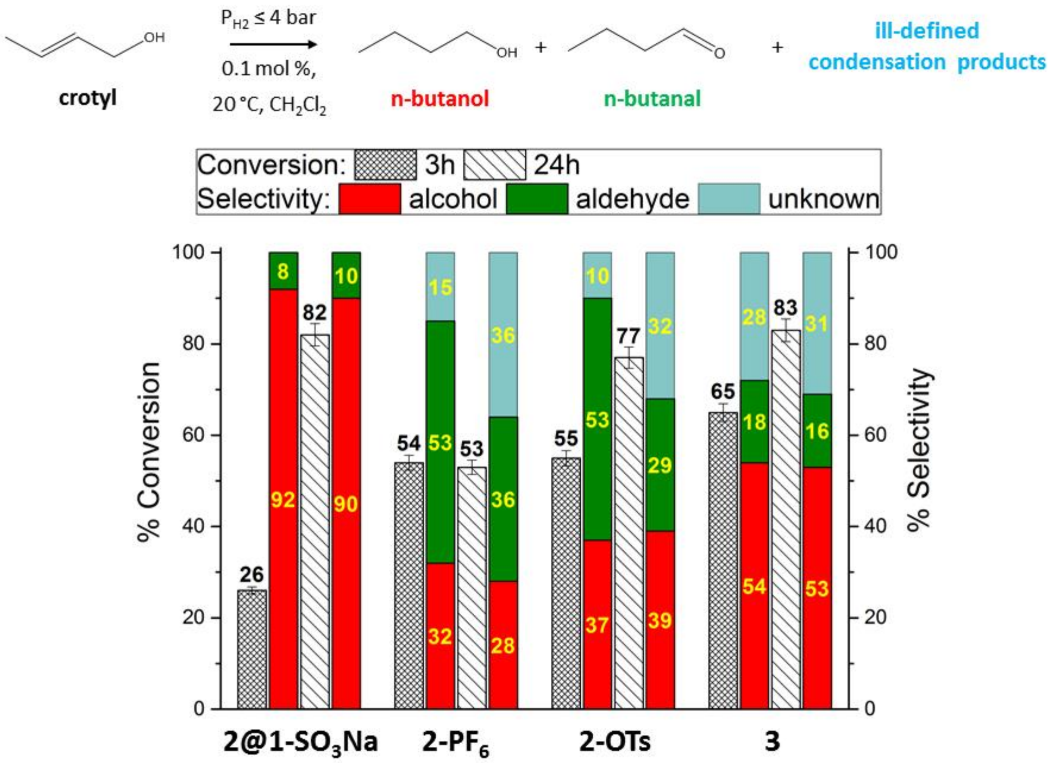

Figure S30. Conversion and selectivity (in colour) for the hydrogenation of crotyl alcohol (**13a**) in 3 h and 24 h with heterogeneous catalyst **2@1-SO₃Na** and homogeneous catalysts **2-PF₆**, **2-OTs** and **3**. Introduction of sulfonate groups prolongs the catalyst's lifetime but does not significantly improve selectivity for the homogeneous systems. Ill-defined condensation byproducts are also detected by ^{1}H NMR and GC for all homogeneous reactions.

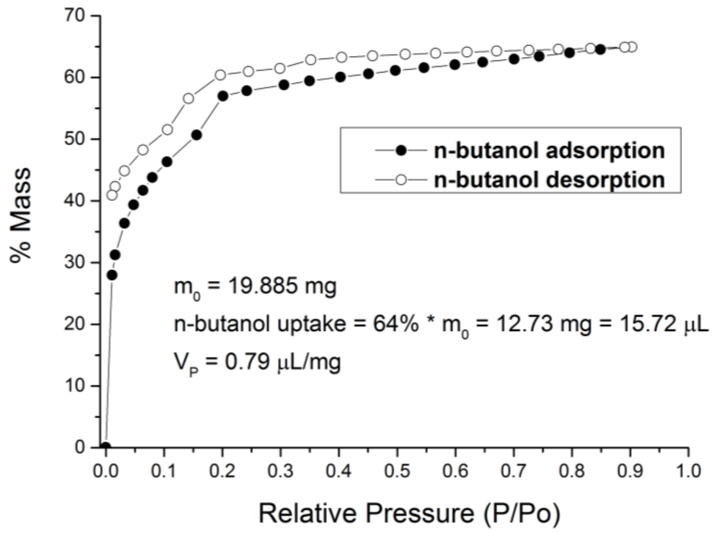

Figure S31. Gravimetric n-butanol uptake by desolvated **1-SO_3H**. Pore volume (V_P) is shown in the inset and lies in excellent agreement with N_2 adsorption-desorption measurements.

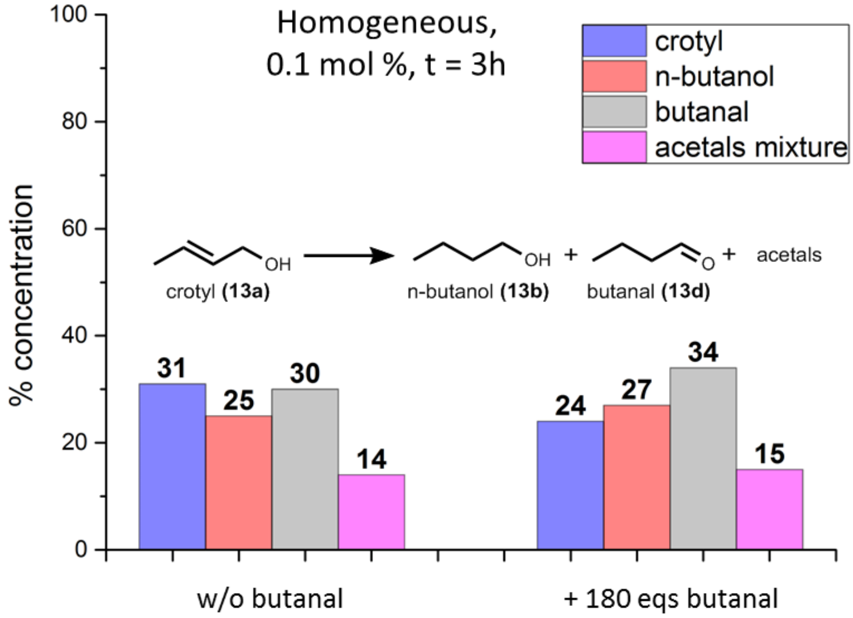

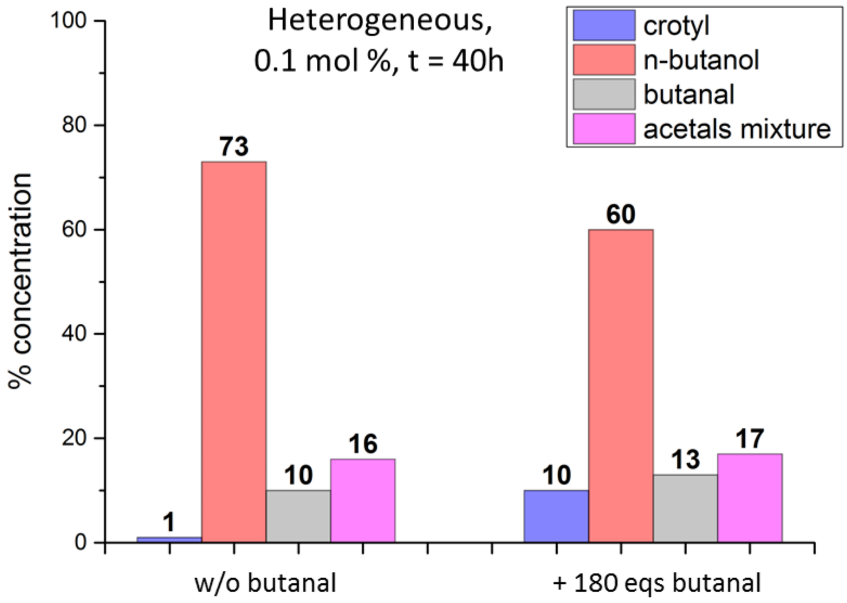

Figure S32. Product inhibition test. Top: Composition of the reaction mixture for the homogeneous system (**2-PF$_6$** as the catalyst) without (left) or with 180 equivalents of butanal added (right). Bottom: Composition of the reaction mixture for the heterogeneous system (**2@1-SO$_3$Na** as the catalyst) without (left) or with the 180 equivalents of butanal added (right). Addition of butanal does not significantly change relative concentration of reagents for either system within experimental error (see also Table S7).

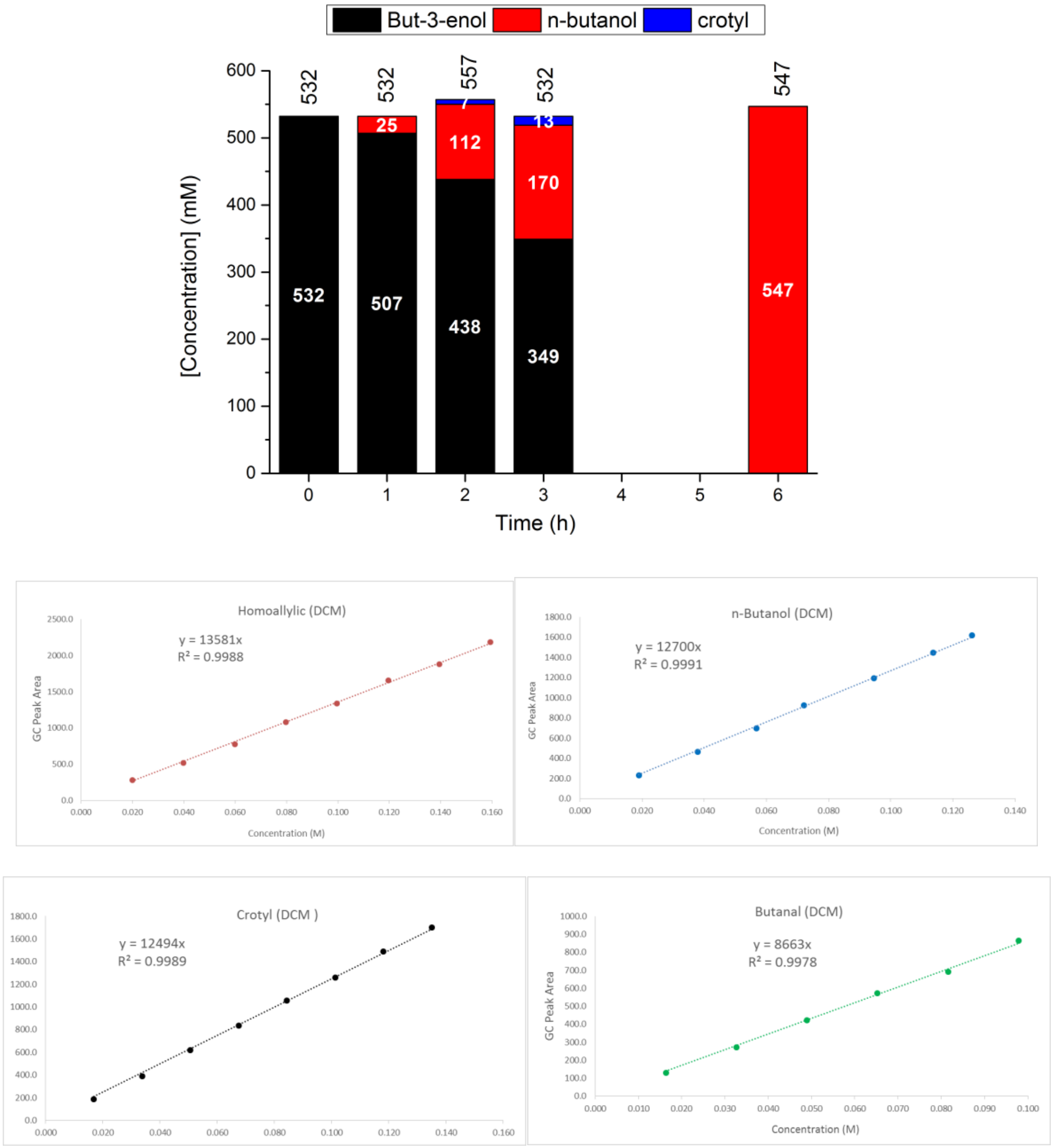

Figure S33. Product distribution based on GC for the hydrogenation of but-3-en-1-ol with **2@1-SO$_3$Na** as the catalyst. Total amount of substrate and products remains constant within experimental error of the GC analysis method (± 5%). Calibration curves for but-3-en-1-ol (top left), butanol (top right), crotyl alcohol (bottom left) and butanal (bottom right) are also shown.

6. References

[1] R. H. Crabtree, J. M. Quirk, H. Felkin, T. Fillebeen-khan, *Synthesis and Reactivity in Inorganic and Metal-Organic Chemistry* **1982**, *12*, 407-413.

[2] R. H. Crabtree, H. Felkin, G. E. Morris, *J. Organonomet. Chem.* **1977**, *141*, 205-215.

[3] a) P. Tarazona, *Phys. Rev. A* **1985**, *32*, 3148-3148; b) P. Tarazona, *Phys. Rev. A* **1985**, *31*, 2672-2679.

[4] A. T. Lubben, J. S. McIndoe, A. S. Weller, *Organometallics* **2008**, *27*, 3303-3306.

[5] S. D. Pike, T. Krämer, N. H. Rees, S. A. Macgregor, A. S. Weller, *Organometallics* **2015**, *34*, 1487-1497.

[6] F. M. Chadwick, A. I. McKay, A. J. Martinez-Martinez, N. H. Rees, T. Kramer, S. A. Macgregor, A. S. Weller, *Chem. Sci.* **2017**, *8*, 6014-6029.

[7] A. Le Bail, *Powder Diffr.* **2005**, *20*, 316-326.

[8] J. Cosier, A. M. Glazer, *J. Appl. Cryst.* **1986**, *19*, 105-107.

[9] Oxford Diffraction Ltd.

[10] G. M. Sheldrick, *Acta Cryst. A* **2015**, *71*, 3-8.

[11] G. M. Sheldrick, *Acta Cryst. A* **2008**, *64*, 112-122.

[12] O. V. Dolomanov, L. J. Bourhis, R. J. Gildea, J. A. K. Howard, H. Puschmann, *J. Appl. Cryst.* **2009**, *42*, 339-341.

[13] G. Akiyama, R. Matsuda, H. Sato, M. Takata, S. Kitagawa, *Adv. Mater.* **2011**, *23*, 3294-3297.

[14] a) J. Juan-Alcaniz, R. Gielisse, A. B. Lago, E. V. Ramos-Fernandez, P. Serra-Crespo, T. Devic, N. Guillou, C. Serre, F. Kapteijn, J. Gascon, *Catal. Sci. Technol.* **2013**, *3*, 2311-2318; b) Y.-X. Zhou, Y.-Z. Chen, Y. Hu, G. Huang, S.-H. Yu, H.-L. Jiang, *Chem. Eur. J.* **2014**, *20*, 14976-14980.

[15] H. M. Lee, T. Jiang, E. D. Stevens, S. P. Nolan, *Organometallics* **2001**, *20*, 1255-1258.

[16] a) G. Férey, C. Mellot-Draznieks, C. Serre, F. Millange, J. Dutour, S. Surblé, I. Margiolaki, *Science* **2005**, *309*, 2040-2042; b) O. I. Lebedev, F. Millange, C. Serre, G. Van Tendeloo, G. Ferey, *Chem. Mater.* **2005**, *17*, 6525-6527.

[17] S. Alvarez, *Dalton Trans.* **2013**, *42*, 8617-8636.
